# Bone Inner Structure Suggests Increasing Aquatic Adaptations in Desmostylia (Mammalia, Afrotheria)

**DOI:** 10.1371/journal.pone.0059146

**Published:** 2013-04-02

**Authors:** Shoji Hayashi, Alexandra Houssaye, Yasuhisa Nakajima, Kentaro Chiba, Tatsuro Ando, Hiroshi Sawamura, Norihisa Inuzuka, Naotomo Kaneko, Tomohiro Osaki

**Affiliations:** 1 Steinmann Institut für Geologie, Paläontologie und Mineralogie, Universität Bonn, Bonn, Germany; 2 Osaka Museum of Natural History, Higashi-sumiyoshi-ku, Osaka, Japan; 3 Laboratory of Dead Body Science, The University Museum, The University of Tokyo, Tokyo, Japan; 4 Division of Earth and Planetary Sciences, Graduate School of Science, Hokkaido University, Sapporo, Japan; 5 Ashoro Museum of Paleontology, Ashoro, Japan; 6 Department of Cell Biology and Anatomy, Graduate School of Medicine, University of Tokyo, Tokyo, Japan; 7 Geological Museum, Geological Survey of Japan, AIST, Ibaraki, Japan; 8 Faculty of Agriculture, School of Veterinary Medicine, University of Tottori, Tottori, Japan; Team 'Evo-Devo of Vertebrate Dentition', France

## Abstract

**Background:**

The paleoecology of desmostylians has been discussed controversially with a general consensus that desmostylians were aquatic or semi-aquatic to some extent. Bone microanatomy can be used as a powerful tool to infer habitat preference of extinct animals. However, bone microanatomical studies of desmostylians are extremely scarce.

**Methodology/Principal Findings:**

We analyzed the histology and microanatomy of several desmostylians using thin-sections and CT scans of ribs, humeri, femora and vertebrae. Comparisons with extant mammals allowed us to better understand the mode of life and evolutionary history of these taxa. Desmostylian ribs and long bones generally lack a medullary cavity. This trait has been interpreted as an aquatic adaptation among amniotes. *Behemotops* and *Paleoparadoxia* show osteosclerosis (i.e. increase in bone compactness), and *Ashoroa* pachyosteosclerosis (i.e. combined increase in bone volume and compactness). Conversely, *Desmostylus* differs from these desmostylians in displaying an osteoporotic-like pattern.

**Conclusions/Significance:**

In living taxa, bone mass increase provides hydrostatic buoyancy and body trim control suitable for poorly efficient swimmers, while wholly spongy bones are associated with hydrodynamic buoyancy control in active swimmers. Our study suggests that all desmostylians had achieved an essentially, if not exclusively, aquatic lifestyle. *Behemotops*, *Paleoparadoxia* and *Ashoroa* are interpreted as shallow water swimmers, either hovering slowly at a preferred depth, or walking on the bottom, and *Desmostylus* as a more active swimmer with a peculiar habitat and feeding strategy within Desmostylia. Therefore, desmostylians are, with cetaceans, the second mammal group showing a shift from bone mass increase to a spongy inner organization of bones in their evolutionary history.

## Introduction

**Figure 1:**
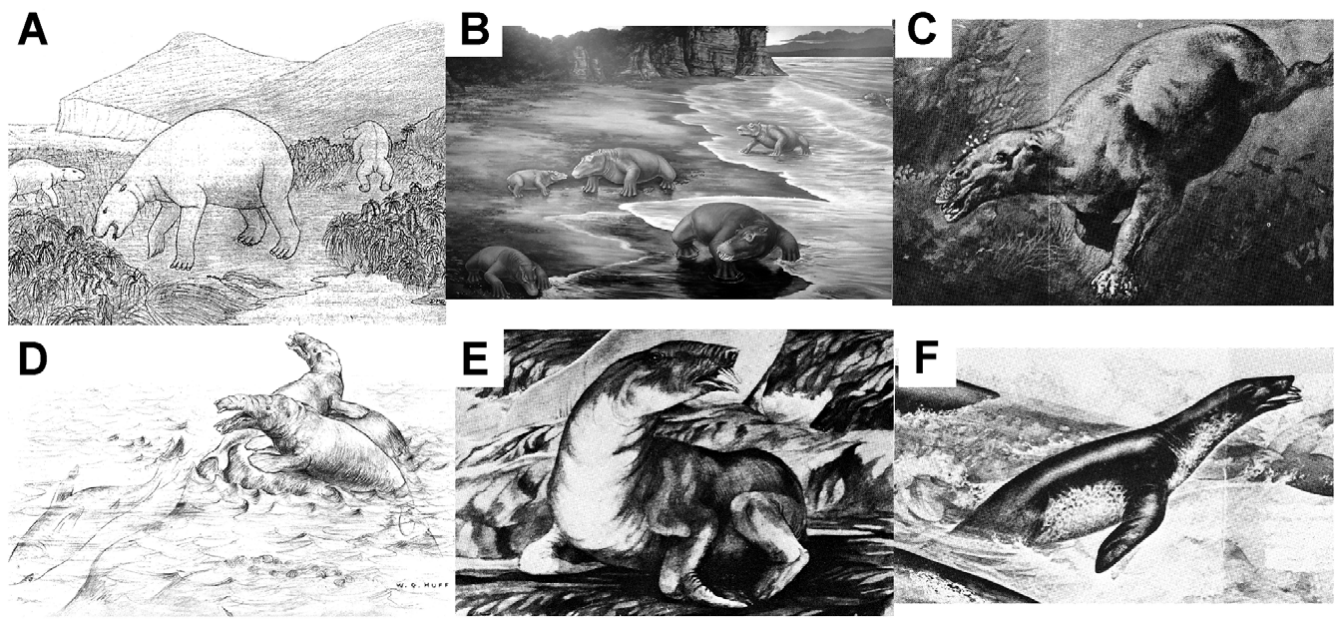
Various restorations of desmostylians based on morphological data illustrating the diverse lifestyles proposed. A, semi-aquatic (like the polar bear) (from [17]). B, bottom wader (from Inuzuka's restoration; the figure is printed with the permission of the Geological Museum, Geological Survey of Japan). C, bottom walker, *Hippopotamus*-like (from [73]); D, bottom swimmer, sirenian-like (from [22]). E–F, active swimmer, pinniped-like (from [23]–[24]).

Desmostylians are a group of extinct mammals known from the Lower Oligocene to the Upper Miocene marine strata of the northern Pacific Rim [1]–[6]. Their paleoecology has remained ‘mysterious’ since their first discovery [7]. Their unique osteological and dental morphologies (e.g. [1], [8]–[14]) have hindered a consensus on their life style. Many researchers have notably discussed the desmostylian semi-aquatic [2], [14]–[17] or essentially aquatic [18]–[19] mode of life, a question of peculiar interest to infer their paleoecology (e.g. diet and locomotion). Previous studies have proposed different reconstructions of their posture [12]–[13], [17], [19]–[20], which resulted in conflicting interpretations on their mode of life. Depending on authors, they have been either referred to as close to that of extinct ground sloths and/or polar bears (Figure 1A; [17]), bears [14], hippopotamids (Figure 1B–C; [12]–[13], [20]–[21]), sirenians (Figure 1D; [22]), or pinnipeds (Figure 1E–F; [11], [18], [23]–[25]).

Bone inner structure is known to be a powerful tool to infer the mode of life of extinct animals, and notably the degree of adaptation to an aquatic life in lineages that secondarily invaded the marine realm (e.g. [26]–[31]). The histology and microanatomy of desmostylians are extremely poorly known despite a few mentions in the literature [8], [30], [32]–[35]. This paper presents the first histological and microanatomical analysis of various desmostylians (*Behemotops*, *Paleoparadoxia*, *Ashoroa* and *Desmostylus*) and discusses their lifestyle, in the light of these new data.

## Materials and Methods

### Materials

**Figure 2:**
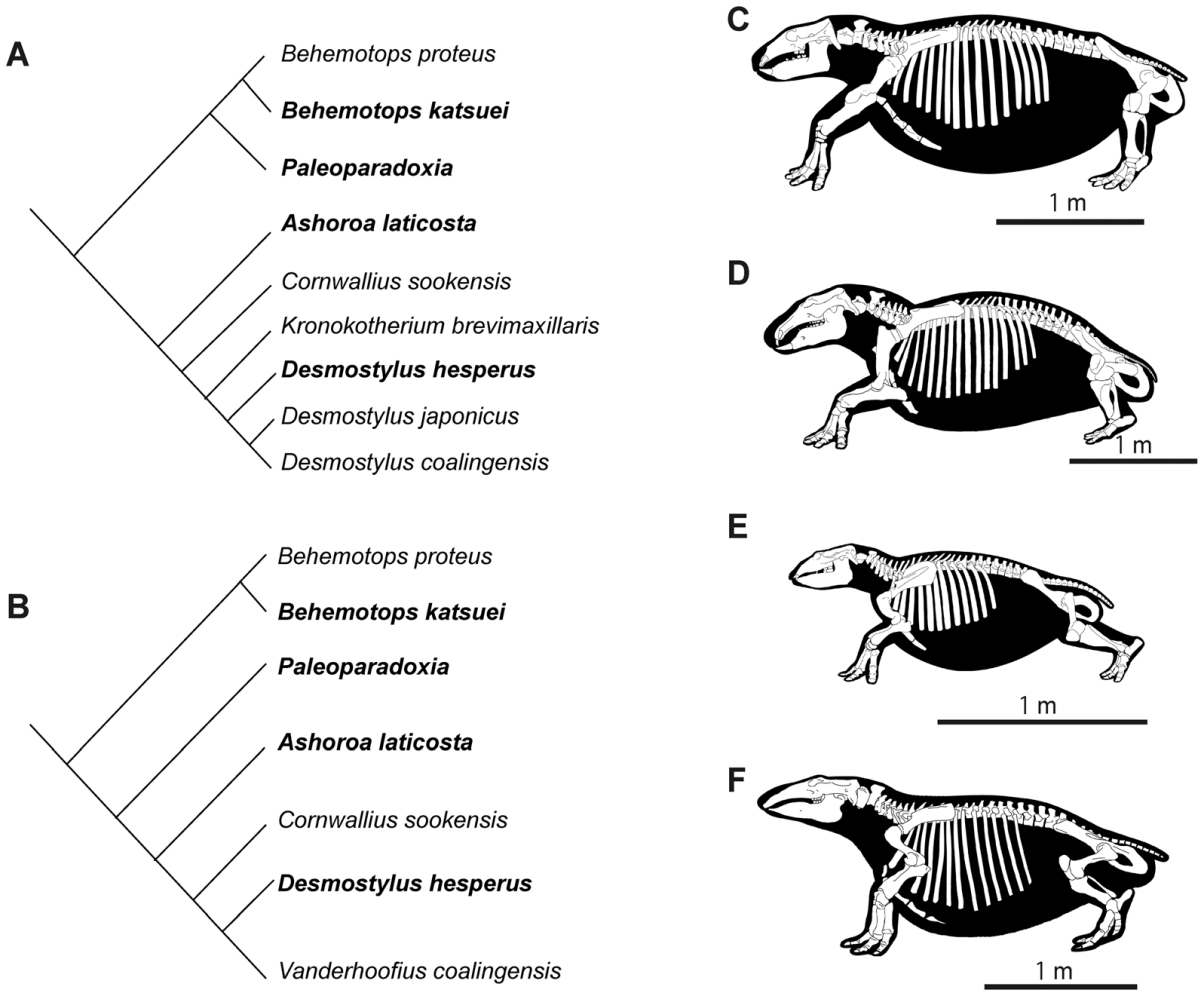
Two phylogenetic hypotheses of desmostylians. A, Phylogenetic hypothesis following Inuzuka 2005 [13]. B, Phylogenetic hypothesis from Beatty 2009 [4]. C–F, skeletal illustrations of various desmostylians. C, *Behemotops*. D, *Paleoparadoxia*. E, *Ashoroa*. F, *Desmostylus*. Illustrations by Tatsuya Shinmura. Studied taxa in bold. Material of *Paleoparadoxia* is not identified at the species level.

Four genera of desmostylians (of the seven known; [4]) were studied for this research (Table 1; see also Figure 2): *Behemotops* [36] (AMP 22 and 52), *Paleoparadoxia* [37] (AMP AK0011, 1001 and 1002), *Ashoroa* [2] (AMP 21/UHR 31990) and *Desmostylus* [7] (GSJ F07743, 07745-4, 07745-7, 07748-1 and UHR18466; see Table 1 for details). Information on institutional abbreviations appearing in the inventor numbers of the concerned specimens is available in the supporting material (Text S1). Eleven ribs, four humeri, five femora and eight vertebrae were analyzed (cf. Table 1). The morphology of most specimens (AMP 21/UHR 31990, AMP 22, GSJ F07743 and UHR 18466) was already well described in the literature [4], [13], [19]–[21], [38]–[39]. One rib (GSJ F07745-4) and one femur (GSJ F07745-7) have not been described yet, but the associated skull was already described in [40]. Other specimens (AMP 52, AMP AK0011, 1001, 1002 and GSJ F07748-1) have not been described at present (see below).

**Table 1:** List of desmostylian specimens.

Species	Abb	Collection number	Part	C	P	S	MD (mm)
***Behemotops katsuiei***	Bk	AMP 22 (Holotype)	Femur▴	N.A.	N.A.	N.A.	72.9
			8^th^ rib^★^▴	0.859	0.088	0.239	37.8
			Fragmentary rib^★^	0.877	0.321	0.125	40.8
			10^th^ thora. vert. ◊	N.A.	N.A.	N.A.	N.A.
			2^nd^ lumber vert. ◊	N.A.	N.A.	N.A.	N.A.
		AMP 52	Isolated ribs▴‡	N.A.	N.A.	N.A.	37.2
***Paleoparadoxia*** ** sp.**	Ps	AMP AK0011	Humerus^★^▴	0.991	0.187	0.056	71.5
		AMP AK1001	Fragmentary rib^★^	0.964	0.123	0.083	42.0
		AMP AK1002	Fragmentary rib^★^	0.888	0.225	0.126	32.0
***Ashoroa laticosta***	Al	AMP 21/UHR 31990 (Holotype)	Humerus▴	N.A.	N.A.	N.A.	47.3
			Femur▴	N.A.	N.A.	N.A.	37.2
			6^th^ rib^★^▴	0.904	0.348	0.002	31.0
			4^th^ thora. vert. ◊	N.A.	N.A.	N.A.	N.A.
			10^th^ thora. vert. ◊	N.A.	N.A.	N.A.	N.A.
			2^nd^ lumber vert. ◊	N.A.	N.A.	N.A.	N.A.
***Desmostylus hesperus***	Dh	UHR18466 (Keton Specimen)	Humerus◊	N.A.	N.A.	N.A.	70.5
			Femur▴	N.A.	N.A.	N.A.	85.7
			5^th^ rib‡	0.611	0.786	0.137	47.2
			6^th^ rib‡◊	0.599	0.719	0.189	38.3
			11^th^ rib‡	0.586	0.845	0.051	37.9
			13^th^ rib‡	0.477	0.809	0.068	38.2
		GSJ F07743 (Utanobori specimens)	Humerus◊	0.464	0.788	0.057	41.0
			4^th^ thora. vert. ◊	N.A.	N.A.	N.A.	N.A.
			10^th^ thora. vert. ◊	N.A.	N.A.	N.A.	N.A.
			2^nd^ lumber vert. ◊	N.A.	N.A.	N.A.	N.A.
		GSJ F07745-4 (Utanobori specimens)	Rib^★^◊	0.633	0.673	0.119	27.8
		GSJ F07745-7 (Utanobori specimens)	Femur^★^◊	0.596	0.631	0.162	>38.0
		GSJ F07748-1	Femur‡	0.690	0.606	0.102	82.6

Bone elements from same specimen number indicates same individual. Abbreviations: ◊, micro CT; ▴, medical CT; ^★^, thin-section; ‡, fracture surface; thora., thoracic; vert., vertebra; N.A., not applicable. Abbreviations for the parameters: C, bone compactness; MD, maximum diameter of cross section; S, width of the transition zone between the cortical bone and the medullary region; P, proportional to the size of the medullary cavity. Abb: list of abbreviations used in Figures 9–11, 13.

*Behemotops* is either a paleoparadoxiid (Figure 2A; [13]) or the most basal desmostylian (Figure 2B; [4]) and has been found in the United States (*Behemotops proteus*) and in Japan (*B. katsuiei*). *Behemotops katsuiei* is a large taxon among desmostylians (estimated body length [EBL] of 290 cm; [13]) known from the marine strata (Upper Morawan Formation) of the Late Oligocene of Ashoro, Hokkaido, Japan [39]. Two ribs, a femur and two vertebrae from the holotype of *Behemotops katsuiei* [39] (AMP 22), as well as thoracic ribs (AMP 52) were histologically examined. The latter, which were found as unassociated bones, were tentatively referred to *Behemotops* sp., despite the absence of diagnostic features in the rib morphology, based on the fact that all the desmostylian fossils discovered from the same locality (Upper Morawan Formation, Ashoro, Hokkaido, Japan) belong to this genus.

*Paleoparadoxia*, one of the largest desmostylians (EBL = 303 cm; [13]), is known from the marine strata of the Middle Miocene of Japan and North America [13]. Two ribs (AMP AK1001 and 1002) and a humerus (AMP AK0011) discovered in the Tonokita Formation (Middle Miocene) in Akan, Hokkaido, Japan, were sampled for this study. These materials were referred to *Paleoparadoxia* sp. since the humerus shows several diagnostic characters of *Paleoparadoxia* [13] – an anteriorly bent lateral epicondylar crest and a small diameter of the humeral trochlea – and since these bones were found in association with a tooth belonging to this genus. The species could not be determined.

*Ashoroa*, the smallest (EBL = 168 cm; [13]) and one of the oldest desmostylians known to date [21], is known from marine strata of the Lower Morawan Formation (early Late Oligocene) in Ashoro, Hokkaido, Japan [21]. The single species of the genus was discovered in Japan. A rib, a humerus, a femur and three vertebrae from the holotype of *Ashoroa laticosta* [21] (AMP 21/UHR 31990) were sampled.

The middle-sized *Desmostylus* (EBL = 271 cm; [13]) is the most derived desmostylid (Figure 2A; [13]) or one of the most derived desmostylians (Figure 2B; [4]). Two species (*Desmostylus hesperus* and *D. japonicus*) have been recognized and the status of a possible third species (*D. coalingensis*) is discussed ([13] contra [4]). *D. hesperus* (and *D. coalingensis*) has(ve) been discovered in North America [1] and both *D. hesperus* and *D. japonicus* in the marine strata of Japan. Most of our sample (five ribs, a humerus, three femora and three vertebrae) were taken from an incomplete skeleton (GSJ F07745, i.e. GSJ F07745-4 and 07745-7) and from two nearly complete skeletons of *Desmostylus hesperus* (UHR 18466 and GSJ F07743), classically referred to as the ‘Utanobori specimens’ (GSJ F07743 and -45; [20], [40]) and the ‘Keton specimen’ (UHR 18466; e.g. [19]–[20], [38]), which are the best-preserved desmostylid fossils in the world. The former was discovered in the Tachikaraushinai Formation, Kamitokushibetsu, Utanobori, Esashi, Hokkaido, and the latter in the Naihoro Coal-bearing Formation (Middle Miocene) in the Keton River, South Sakhalin. Additionally, an isolated femur (GSJ F07748-1) from the Tachikaraushinai Formation was histologically examined. This bone was referred to *Desmostylus* cf. *hesperus* as it shows several diagnostic characters of the genus *Desmostylus*: a flat femoral shaft, a short femoral neck, a strong anterior projection of the greater trochanter, a distal position of the lesser trochanter and a shallow trochanteric groove [13], and because it was found in the same locality as other bones referred to this species.

Ontogenetic stages of our samples (except the material that was too fragmentary) were determined based on teeth, neurocentral sutures of vertebrae and possible occurrence of epiphyseal fusions of long bones (c.f. [41]). Deciduous teeth and neurocentral sutures in vertebrae and epiphyseal fusions in long bones are absent in *Paleoparadoxia* AMP 0011, AMP AK1001, 1002, *Ashoroa* AMP 21/UHR 31990, and *Desmostylus* UHR 18466, GSJ F07748-1, while neurocentral sutures of vertebrae and epiphyseal fusions on long bones are present in *Behemotops* AMP 22 and *Desmostylus* GSJ F07743, 07745 (i.e. GSJ F7745-4 and -7). In *Behemotops* AMP 22, third molars (M3) are present. Therefore, the former were referred to as adults and *Behemotops* AMP 22 as a subadult, and the others as juveniles. Only the ontogenetic status of AMP 52, which only comprises unassociated ribs, could not be determined.

For comparative purposes, ribs from 19 mammal taxa (18 extant and one extinct), humeri from 62 extant mammal taxa, femora from 16 extant mammal taxa and vertebrae from 11 extant mammal taxa with various phylogenic positions and ecologies were examined (Tables 2–5).

**Table 2:** List of comparative rib specimens.

Order	Species	Abb	Common name	E	Collection number	C	P	S	MD (mm)
**Sirenia**	*Trichechus manatus*	Tm	Manatee	PA	NSM M 34694^★^	0.994	0*	0*	77.8
	*Halitherium schinzii* †	Hs	N.A.	PA	IPB M2384^★^	0.980	0*	0*	50.0
**Hyracoidea**	*Procavia capensis*	Pc	Rock hyrax	T	NSM M 34971◊	0.638	0.599	0.020	2.5
**Tubulidentata**	*Orycteropus afer*	Ora	Aardvark	T	NSM M 34334◊	0.563	0.648	0.040	8.8
**Artiodactyla**	*Capra aegagrus*	Ca	Goat	T	UFGK unnumbered◊	0.758	0.587	0.113	11.8
	*Rangifer tarandus*	Rt	Caribou	T	IPB M47◊	0.720	0509	0.083	19.2
	*Ovis aries*	Ova	Sheep	T	UFGK unnumbered◊	0.617	0.764	0.107	15.2
	*Hippopotamus amphibius*	Ha	Hippopotamus	SA	AMP R22▴	0.731	0.499	0.072	39.6
**Perissodactyla**	*Equus caballus*	Ec	Horse	T	UFGK unnumbered◊	0.747	0.653	0.142	36.3
**Carnivora (Pinnipedia excluded)**	*Meles meles*	Mm	European badger	T	IPB M4002◊	0.719	0.637	0.060	7.6
	*Martes foina*	Mf	Beech marten	T	IPB M4004◊	0.691	0.501	0.138	2.2
				T	IPB M319◊	0.691	0.501	0.138	2.0
	*Canis lupus familiaris*	Cl	Dog	T	UFGK unnumbered◊	0.805	0.738	0.051	9.9
	*Tremarctos ornatus*	To	Spectacled bear	T	ZFMK 97.275◊	0.757	0.635	0.022	13.0
	*Ursus maritimus*	Um	Polar bear	SA	ZFMK 2005.356◊	0.690	0.616	0.085	12.2
**Pinnipedia**	*Phoca vitulina*	Pv	Harbor seal	D	IPB M 60	0.469	0.761	0.106	7.8
	*Zalophus californianus*	Zc	California Sea lion	D	ZFMK 49.98◊	0.556	0.736	0.135	13.7
	*Mirounga leonina*	Ml	Elephant seal	D	ZFMK 62.105◊	0.303	0.888	0.092	25.3
**Rodentia**	*Castor fiber*	Cf	Beaver	SA	IPB M2◊	0.820	0.401	0.063	4.7
**Cetacea**	*Balaenoptera brydei*	Bb	Bryde's whale	D	NSM M 32599^★^	0.665	0.651	0.118	45.8

Bone elements from same specimen number indicates same individual. Abbreviations: ◊, micro CT; ▴, medical CT; ^★^, thin-section; †, extinct species; *, original meaningless values set at 0 to conceptualize these osteosclerotic bones as having an infinitely-small medullary cavity and abrupt transition (Laurin, per. comm. 2012); N.A., not applicable. Abb: list of abbreviations used in Figure 9. E: ecological categories; T: terrestrial; SA: semi-aquatic shallow swimmers or divers; PA: exclusively aquatic poorly active swimmers; D: essentially or exclusively aquatic deep divers.

**Table 3:** List of comparative humerus specimens.

Order	Species	Abb	Common name	E	Collectionnumber	C	P	S	MD (mm)
**Sirenia**	*Trichechus manatus*	Tm	Manatee	PA	NSM M 34694^★^	0.997	0*	0*	43.7
	*Dugong dugon*	Dud	Dugong	PA	Laurin et al. (2011)	0.975	0*	0*	26.7
**Hyracoidea**	*Procavia capensis*	Pc	Rock hyrax	T	NSM M 34971◊	0.526	0.685	0.035	9.8
**Tubulidentata**	*Orycteropus afer*	Ora	Aardvark	T	NSM M 34334◊	0.538	0.682	0.032	19.3
**Artiodactyla**	*Hippopotamus amphibius*	Ha	Hippopotamus	SA	AMP R22▴	0.716	0.526	0.041	96.8
	*Sus scrofa*	Ss	Domestic pig	T	Laurin et al. (2011)	0.671	0.569	0.016	20.6
	*Capra falconeri*	Caf	Markhor	T		0.498	0.703	0.018	28.4
	*Ovis ammon*	Ova	Sheep	T		0.514	0.695	0.021	15.6
	*Ammotragus lervia*	Aml	Barbary Sheep	T		0.58	0.639	0.027	25.0
	*Antilope cervicapra*	Ac	Blackbuck	T		0.509	0.694	0.025	15.8
	*Redunca fulvorufula*	Ref	Mountain reedbuck	T		0.699	0.542	0.019	16.4
	*Kobus leche*	Kl	Lechwe	T		0.582	0.641	0.022	24.7
	*Bison bison*	Bb	American bison	T		0.701	0.54	0.012	61.0
	*Taurotragus oryx*	To	Common eland	T		0.659	0.534	0.048	43.1
	*Boselaphus tragocamelus*	Bt	Nilgai	T		0.787	0.454	0.022	36.8
	*Rangifer tarandus*	Rt	Caribou	T		0.599	0.626	0.033	22.6
	*Capreolus capreolus*	Cc	Roe deer	T		0.625	0.604	0.021	10.1
	*Cervus elaphus*	Ce	Red deer	T		0.627	0.605	0.023	28.8
	*Dama dama*	Dad	Fallow deer	T		0.642	0.593	0.021	21.7
	*Axis axis*	Axa	Chital	T		0.633	0.602	0.019	22.0
	*Syncerus caffer*	Sc	African buffalo	T		0.686	0.549	0.025	45.9
**Perissodactyla**	*Equus burchelli*	Eb	Plains zebra	T		0.736	0.51	0.021	44.2
**Carnivora (Pinnipedia excluded)**	*Meles meles*	Mm	European badger	T	UMUT 08361◊	0.524	0.683	0.023	11.5
	*Canis lupus*	Cl	Gray wolf	T	Laurin et al. (2011)	0.672	0.561	0.02	21.1
	*Panthera leo*	Pnl	Lion	T		0.56	0.654	0.05	42.6
	*Vulpes vulpes*	Vv	Red fox	T	NSM M 36987◊	0.611	0.624	0.012	8.8
	*Procyon lotor*	Prl	Raccoon	T	NSM M 34935◊	0.642	0.592	0.030	12.9
	*Prionailurus bengalensis*	Pb	Leopard cat	T	NSM M 19834◊	0.546	0.673	0.012	8.2
	*Acinonyx jubatus*	Aj	Cheetah	T	NSM M 37279◊	0.542	0.674	0.020	22.9
	*Uncia uncia*	Uc	Snow leopard	T	NSM M 33876◊	0.625	0.611	0.020	20.7
	*Nyctereutes procyonoides*	Np	Racoon dog	T	NSM M 37371◊	0.535	0.691	0.023	9.0
	*Gulo gulo*	Gg	Wolverine	T	NSM M 33044◊	0.695	0.549	0.026	12.8
	*Paguma larvata*	Pal	Masked palm civet	T	NSM M 36806◊	0.678	0.563	0.021	9.2
	*Amblonyx cinereus*	Amc	Oriental small-clawed-otter	SA	Laurin et al. (2011)	0.649	0.589	0.059	5.3
	*Lutra lutra*	Ll	European otter	SA	NSM M 16201◊	0.792	0.554	0.063	14.2
	*Enhydra lutris*	El	Sea otter	SA	UMUT 12247◊	0.801	0.437	0.052	19.9
	*Ursus thibetanus*	Ut	Asian black bear	T	NSM M 35844◊	0.612	0.616	0.022	31.3
	*Ursus maritimus*	Um	Polar bear	SA	ZFMK 2005.356◊	0.389	0.779	0.041	40.0
**Pinnipedia**	*Phoca sibirica*	Ps	Baikal seal	D	NSM M 29710◊	0.630	0.652	0.099	31.5
	*Phoca caspica*	Phc	Caspian seal	D	NSM M 30044◊	0.833	0.452	0.061	24.3
	*Zalophus californianus*	Zc	California Sea lion	D	NSM M 29641◊	0.772	0.796	0.005	53.0
	*Otaria flavescens*	Of	South American sea lion	D	NSM M 29890◊	0.732	0.754	0.043	39.3
	*Callorhinus ursinus*	Cu	Northern fur seal	D	NSM M 29642◊	0.766	0.814	0.100	48.0
	*Arctocephalus australis*	Aa	South American fur seal	D	Laurin et al. (2011)	0.808	0.635	0.068	43.4
	*Mirounga leonina*	Ml	Elephant seal	D		0.404	0.828.	0.107	72.1
	*Leptonychotes weddelli*	Lw	Weddell seal	D	NSM M 29643◊	0.763	0.721	0.091	48.6
**Rodentia**	*Myocastor coypus*	Mc	Coypu	SA	Laurin et al. (2011)	0.814	0.446	0.077	9.2
	*Ondatra zibethicus*	Oz	Muskrat	SA		0.825	0.411	0.031	4.3
	*Cavia porcellus*	Cp	Guinea pig	T		0.757	0.49	0.079	3.7
	*Marmota marmota*	Mam	Alpine marmot	T		0.567	0.67	0.022	7.5
	*Hydrochoerus hydrochaeris*	Hh	Capybara	SA		0.599	0.626	0.044	18.5
	*Hystrix cristata*	Hc	Crested porcupine	T		0.735	0.505	0.04	12.3
**Erinaceidae**	*Erinaceus europaeus*	Ee	European hedgehog	T		0.611	0.666	0.03	4.8
**Macropodidae**	*Macropus rufogriseus*	Mr	Red-necked wallaby	T		0.599	0.632	0.025	10.5
				T		0.774	0.466	0.052	12.5
**Dasypodidae**	*Zaedyus pichiy*	Zp	Dwarf armadillo	T		0.849	0.397	0.091	6.9
**Talpidae**	*Galemys pyrenaicus*	Gp	Pyrenean desman	SA		0.801	0.441	0.027	1.9
**Ornithorhynchidae**	*Ornithorhynchus anatinus*	Oa	Platypus	SA		0.88	0.289	0.134	7.5
**Solenodontidae**	*Solenodon paradoxus*	Sp	Hispaniolan solenodon	T		0.531	0.676	0.063	6.3
**Cercopithecidae**	*Macaca radiata*	Mr	Bonnet macaque	T		0.584	0.633	0.048	9.1
	*Chlorocebus aethiops*	Cha	Grivet	T		0.681	0.545	0.036	12.1
**Cetacea**	*Delphinus delphis*	Dd	Short-beaked common dolphin	D		0.562	0.866	0.151	30.5
	*Neophocaena phocaenoides*	Nep	Finless porpoise	D	NSM M unnumbered◊	0.631	0.908	0.048	23.1

Bone elements from same specimen number indicates same individual. Abbreviations: ◊, micro CT; ▴, medical CT; ^★^, thin-section; *, original meaningless values set at 0 to conceptualize these osteosclerotic bones as having an infinitely-small medullary cavity and abrupt transition (Laurin, per. comm. 2012); N.A., not applicable. Abb: list of abbreviations used in Figure 10. The microanatomical data of some taxa were calculated based on the figures of [74]. E: list of categories as in Table 2.

**Table 4:** List of comparative femur specimens.

Order	Species	Abb	Common name	E	Collection number	C	P	S	MD (mm)
**Artiodactyla**	*Hippopotamus amphibius*	Ha	Hippopotamus	SA	AMP R22▴	0.794	0.448	0.022	69.7
	*Rangifer tarandus*	Rt	Caribou	T	IPB M47◊	0.441	0.742	0.014	29.0
	*Lama guanicoe*	Lg	Guanaco	T	IPM M7388◊	0.461	0.727	0.024	30.3
	*Sus scrofa*	Ss	Domestic pig	T	IPM M56	0.564	0.655	0.014	23.8
	*Capreolus capreolus*	Cc	Roe deer	T	IPM M1452	0.479	0.716	0.010	14.3
**Carnivora (Pinnipedia excluded)**	*Meles meles*	Mm	European badger	T	IPB M4002◊	0.559	0.654	0.020	11.5
	*Vulpes vulpes*	Vv	Red fox	T	IPB M12	0.553	0.678	0.017	10.4
	*Procyon lotor*	Prl	Raccoon	T	NSM M 34935◊	0.66	0.576	0.016	12.7
	*Uncia uncia*	Uc	Snow leopard	T	NSM M 33876◊	0.647	0.592	0.012	20.1
	*Nyctereutes procyonoides*	Np	Raccoon dog	T	NSM M 37331◊	0.631	0.603	0.024	8.1
	*Paguma larvata*	Pal	Masked palm civet	T	NSM M 36806◊	0.656	0.579	0.014	9.3
	*Ursus maritimus*	Um	Polar bear	SA	ZFMK 2005.356◊	0.603	0.620	0.037	32.5
**Pinnipedia**	*Zalophus californianus*	Zc	California Sea lion	D	NSM M 29641◊	0.817	0.439	0.071	25.5
	*Phoca vitulina*	Pv	Harbor seal	D	IPB M 60	0.574	0.700.	0.063	22.0
	*Leptonychotes weddelli*	Lw	Weddell seal	D	NSM M 29643◊	0.66	0.662	0.054	44.9
**Rodentia**	*Castor fiber*	Cf	Beaver	SA	IPB M2◊	0.742	0.488	0.066	29.0

Bone elements from same specimen number indicates same individual. Abbreviations: ◊, micro CT; ▴, medical CT; N.A., not applicable; Abb: list of abbreviations used in Figure 11. E: list of categories as in Table 2.

**Table 5:** List of comparative vertebrate specimens.

Order	Species	Common name	Collection number
**Sirenia**	*Trichechus manatus*	Manatee	ZFMK 73.223◊
**Artiodactyla**	*Rangifer tarandus*	Caribou	IPB Ma47◊
	*Choeropsis liberiensis*	Pygmy hippopotamus	ZFMK 65.570◊
	*Hippopotamus amphibius*	Hippopotamus	AMP R22◊
**Carnivora (Pinnipedia excluded)**	*Tremarctos ornatus*	Spectacled bear	ZFMK 97.275◊
	*Ursus maritimus*	Polar bear	ZFMK 2005.356◊
	*Panthera leo*	Lion	ZFMK 2006.031◊
**Pinnipedia**	*Phoca vitulina*	Harbor seal	IPB M 60◊
	*Zalophus californianus*	California sea lion	ZFMK 49.98◊
	*Mirounga leonina*	Elephant seal	ZFMK 62.105◊
**Rodentia**	*Castor fiber*	Beaver	ZFMK 2006.607 Bone elements from same specimen number indicates same individual. Abbreviations: ◊, micro CT. ◊

All necessary permits were obtained for the described field studies. We obtained permissions from the various museums/institutions (i.e. AMP, GSJ, IPB, NSM, UFGK, UHR, UMUT and ZFMK) to access the collections. All fossil specimens were collected by the respective museums/institutions, and all extant specimens stored at these museums/institutions were donated by zoos and/or aquariums.

### Methods

Almost all specimens (both desmostylians and comparative material; see Tables 1–5) were scanned using either a medical helical CT scanner (RADX-PRATICO, 0.6 mm resolution, 120 kV, 30 mA) at the Graduate School of Veterinary Medicine, Hokkaido University (Japan) or a high-resolution helical CT scanner (GEphoenix∣X-ray v∣tome∣xs, 28–200 µm resolution, 180 kV, 120 mA) at the Institute for Paleontology, University of Bonn (Germany). Image segmentation and visualization were performed using VG-Studio Max (Volume Graphics) version 2.0.

**Figure 3:**
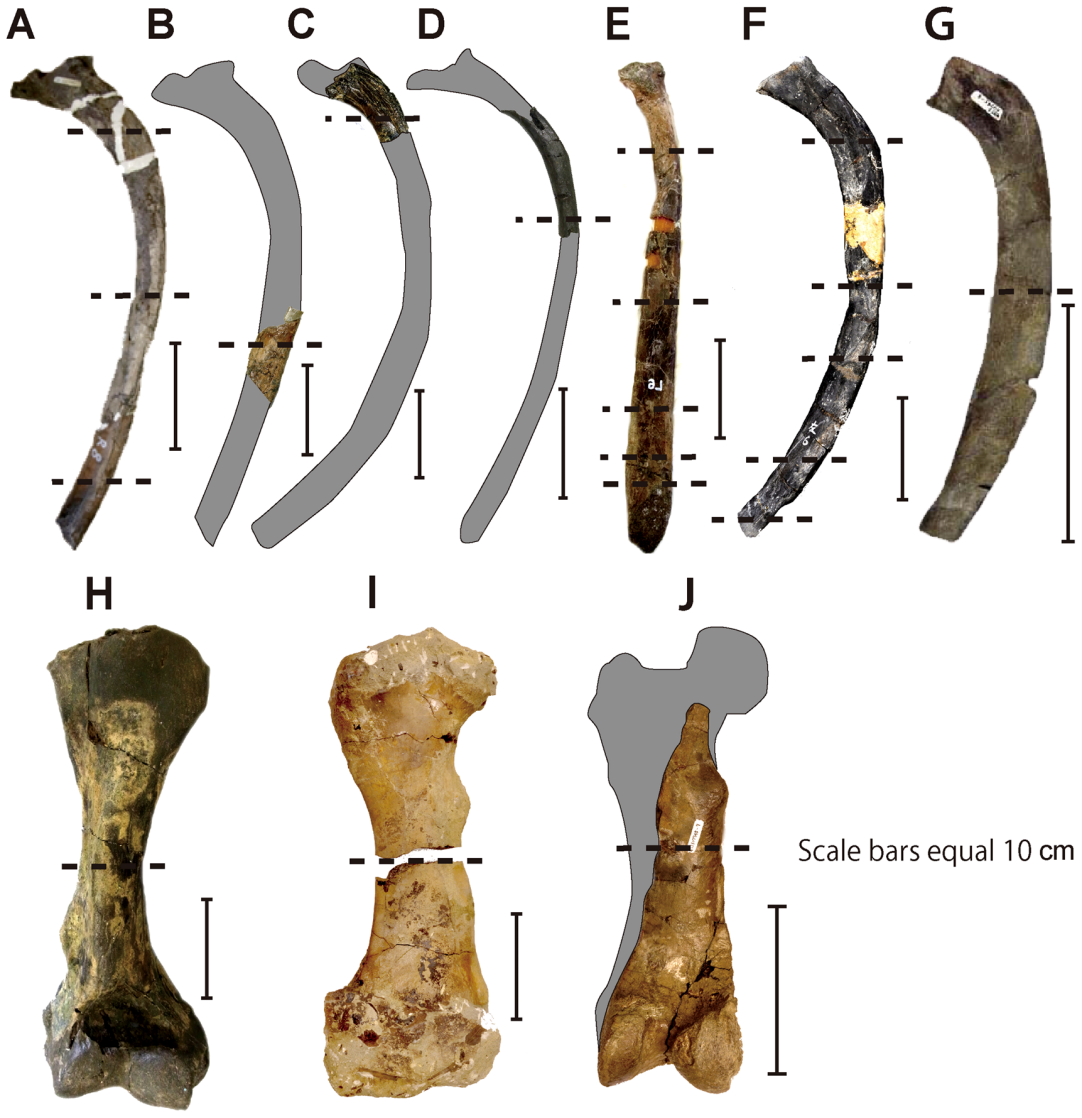
Desmostylian material sectioned in this study (ribs, humeri and femora). A–G. ribs in lateral view. H–J, limb bones in caudal view. A–B, *Behemotops katsuiei*. AMP 22. C–D, *Paleoparadoxia* sp. AMP AK1001 (C) and AMP AK1002 (D). E, *Ashoroa laticosta* AMP 21/UHR 31990. F–G, *Desmostylus hesperus* UHR 18466 (F) and GSJ F07745-4 (G). H, *Paleoparadoxia* sp. humerus AMP AK0011. I–J, *Desmostylus hesperus* femora GSJ F07748-1 (I) and GSJ F07745-4 (J). Sectional planes are represented by dashed lines.

Thin-sections and/or polished sections were made based on the methodology described in [42] and [43]. Prior to sectioning, all desmostylian specimens were photographed and standard measurements were taken. Thin-sections were taken at mid-shaft for long bones and at about mid-length for the ribs (cf. Figure 3). For ribs, additional thin-sections were taken in proximal and distal parts for desmostylian taxa to examine variations along the bone (Figure 3A–F). Thin-sections were photographed with a digital film scanner (Canon Pixus Mp 800) and analyzed with Leica DMLP and Nikon Optiphot2-pol microscopes. Microscopic photos were taken with a Nikon Coolpix 5000. Fracture surfaces of four ribs (UHR 18466) and of a polished section of a femur (GSJ F07748-1) of *Desmostylus* were examined. Quantifications and the analysis of the distribution of bone density were calculated using the software Bone Profiler [44]. We examined three parameters provided by this software to show the bone density distribution: C, P and S. C is the global bone compactness for the whole sectional area. P is the relative distance from the centre of the section to the point of inflection, where the most abrupt change in compactness is observed. P is thus proportional to the size of the medullary cavity. S is the reciprocal of the slope at the inflection point and generally reflects the width of the transition zone between the cortical bone and the medullary region (see details in [44]). Additionally, maximal diameter (MD) of each cross section was measured.

A Principal Component Analysis (PCA) was performed on the parameters cited above using the statistic software R (http://www.r-project.org/). A Linear Discriminant Analysis (LDA) was also performed, in order to infer the lifestyle of the desmostylians based on our comparative material, following the methodology described in [45].

The phylogenetic significance of these parameters was tested for the three skeletal elements (rib, humerus and femur). Random tree generation was used in MESQUITE [46] following the method described in [45]. The taxa were incorporated into a consensual phylogenetic tree (consistent with [4], [47]–[51]; also see supplementary information in Text S2–4). The number of steps for the three characters were analyzed and compared to that obtained for 9999 trees generated by randomization of terminal taxa. The number of trees (random and reference) at least as short as the reference tree divided by 10000 gave the probability that the character does not show any phylogenetic signal (H0). H0 is rejected when this number is less than 5% and the phylogenetic signal is thus considered significant.

**Figure 4:**
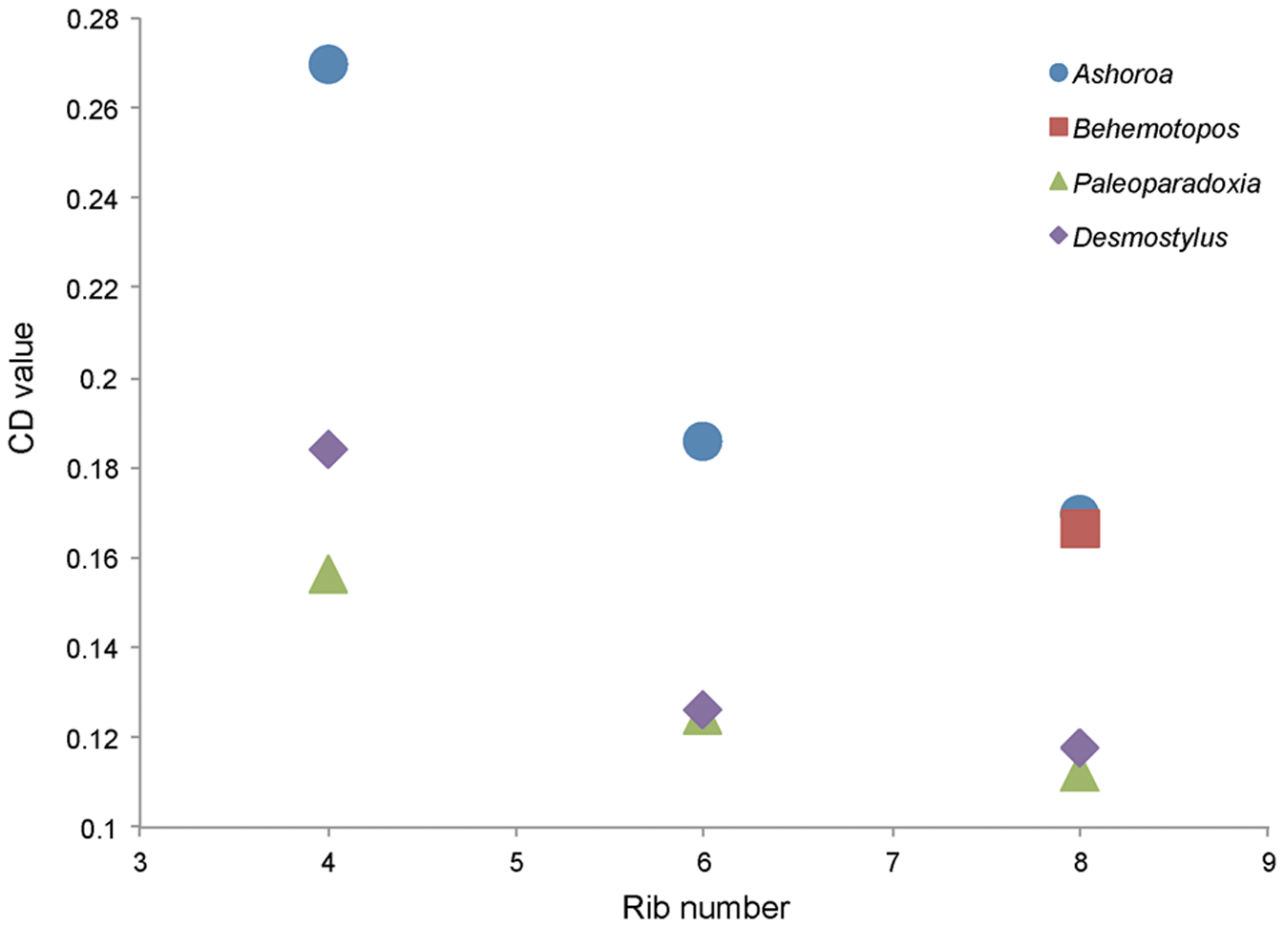
Graph illustrating the cortical development index (CD) in desmostylian ribs. CD is significantly higher in the ribs of *Ashoroa* (0.16–0.26) than in those of the other desmostylians (0.11–0.18). Buffrénil et al. (2010) [30] previously reported that CD values higher than 0.18 characterize a pachyostotic condition. *Ashoroa* ribs (at least from the 4 th to the 6 th thoracic ones) can therefore be considered pachyostotic. In each desmostylian skeleton, the CD value decreases antero-posteriorly.

Gross morphometric data of ribs were measured (Figure 4) in order to quantify the relative development of their periosteal cortices and to assess, on a comparative basis, the possible occurrence of pachyostosis (i.e. increase in bone volume morphologically observable), based on the methodology described in [30]. Two parameters were measured for this purpose: 1) Rib length, an index corresponding to the sum of rib chord + mean rib arrow (i.e. mean of the length of two vectors projected perpendicularly from the chord to the inner and outer rib surfaces at maximum rib bend) and 2) Rib mean circumference, measured at the proximal, middle, and distal thirds of the bone. Cortical development index (CD) corresponds to the division of rib mean circumference by rib length (see [30]). The 6^th^ to 8^th^ ribs were analyzed for each desmostylian taxon (except *Behemotops*; see below) to evaluate the CD values. In *Behemotops*, only the 8^th^ rib was examined because ribs anterior to this one are not known so far (see also [39]). The morphological data of *Paleoparadoxia* ribs were taken based on [13].

## Results

### (a) Qualitative Analysis

#### Desmostylians. Behemotops (Figure 5)

**Figure 5:**
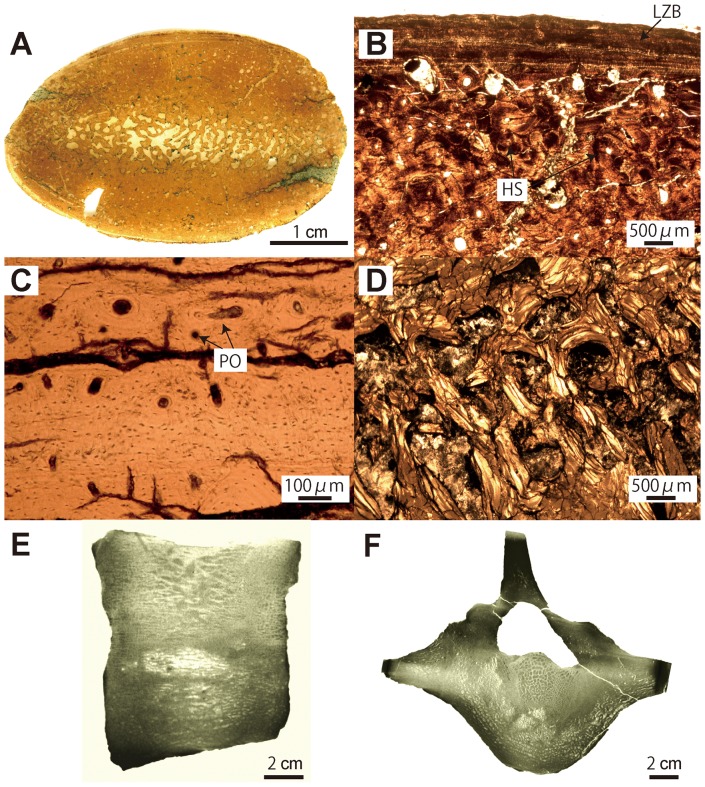
*Behemotops katsuiei* histological and microanatomical features (rib and vertebra: AMP 22). A–D, mid-shaft section of 8^th^ rib. A, whole section. B, cortex in natural light (NL); periphery of the bone at the top. LZB; lamellar zonal bone. HS; Haversian system. C, detail of the LZB in NL. PO; primary osteons. D, trabeculae in the core of the section in polarized light (PL). E–F, 2^nd^ lumbar vertebra. E, virtual mid-sagittal section of the centrum. F, transverse section.

Ribs display a particularly dense inner organization with an extremely compact and thick cortex and a compacted medullary region. There is no open medullary cavity but rather a relatively narrow zone (the pseudo-medullary cavity) with several irregularly shaped cavities separated by thick trabeculae (Figure 5A). The outer part is rather thin consisting of parallel-fibered bone tissue with multiple LAGs (lines of arrested growth; it corresponds to lamellar-zonal bone [LZB]) and with a moderate degree of vascularization consisting of longitudinally-oriented primary osteons (Figure 5B–C). The inner part consists of Haversian bone with no remnant of primary bone (Figure 5B). In the proximal section of a rib, all regions are completely remodeled except the outermost cortex on the lateral side. In the mid-shaft section, while the lateral side exhibits completely remodeled bone, the medial side still shows primary bone (parallel-fibred; see above). In the medullary region, the trabecular struts are completely remodeled and thickened by endosteal deposits (Figure 5D). Many Sharpey's fibers directed perpendicularly to the periosteal surface are present in the outermost cortex. The vascular spaces (i.e. lumen) of the secondary osteons are notably small, due to substantial endosteal deposits, which are slightly more limited in the proximal section. As a result of the increase in endosteal bone deposits, cortical compactness increases from the proximal to the distal part of the rib, and the widths of the cavities in the pseudo-medullary cavity decrease.

Despite the poor resolution of the femur virtual section, this bone also appears very compact, but seems to show a small open medullary cavity.

The vertebrae are cancellous (Figure 5E–F). They consist almost exclusively of spongiosa, the outer layer of compact cortical bone being thin. This spongiosa consists of a tight network of thin trabeculae with small intertrabecular spaces. It is much tighter in the endosteo-endochondral than in the periosteal territory. The trabeculae are antero-posteriorly oriented; they also display a circumferential orientation, except in the innermost part of the bone (Figure 5E–F).

#### Paleoparadoxia (Figure 6)

**Figure 6:**
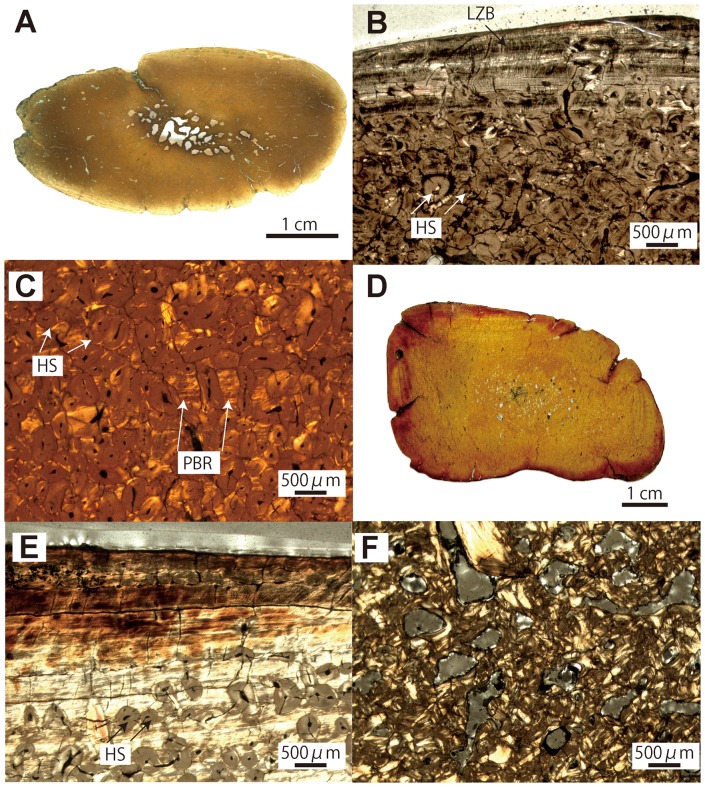
*Paleoparadoxia* sp. histological and microanatomical features (rib: AMP AK1001; humerus: AMP AK0011). A–C, mid-shaft section of fragmentary rib. A, whole section. B, cortex in natural light (NL). C, detail of the cortex of the matrix of cortical bone in polarized light (PL). D–F. mid-shaft section of humerus. D, whole section. E, cortex in PL. F, trabeculae in the core of the section. Periphery of the bone at the top. PBR; primary bone remnant.

Thin-sections of the ribs and of the humerus both show a very compact inner organization (Figure 6A, D). The cortices are almost entirely compact. The medullary region only consists of small cavities (especially in the humerus) separated by thick trabecular struts (Figure 6A, D, F). The histological features of the cortex are similar to those of *Behemotops* (Figure 6B–C, E). Both simple vascular canals and primary osteons occur, but simple vascular canals are dominant. The vascular network shows a longitudinal organization in both the ribs and the humerus (Figure 6B, E). Generally, vascularization is rather abundant, especially in the ribs.

#### Ashoroa laticosta (Figure 7)

The rib morphology of *Ashoroa* differs from that of other desmostylians (Figures 3, 4; see also [21]). It is much broader, especially distally, and evokes sirenian ribs.

**Figure 7:**
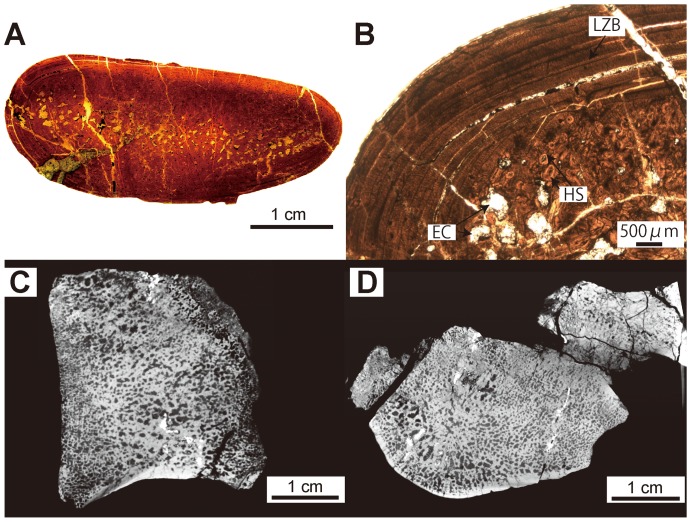
*Ashoroa laticosta* histological and microanatomical features (rib and vertebra: AMP 21/UHR 31990). A–B, mid-shaft section of 6^th^ rib. A; whole section. B, cortex in natural light (NL); periphery of the bone at the top. EC; erosion cavity. C–D, 4^th^ thoracic vertebra. C, virtual mid-sagittal section of the centrum. D, transverse section.

Ribs are very dense with no true medullary cavity, like those of *Behemotops* and *Paleoparadoxia* (Figure 7A). The lamellar-zonal bone tissue (LZB) is only poorly vascularized (Figure 7B), with a few primary osteons and simple vascular canals longitudinally oriented. In all sections, the thickness of the cortex is not homogenous in the whole section. The lateral side is thicker, suggesting an osseous drift. Many erosion cavities occur in the inner cortex in the distal rib section (Figure 7B). This indicates that remodeling was still active in the inner cortex. The CT images of the humerus and femur of AMP 21/UHR 31990 show that these bones lack any large medullary cavities, as seen in *Paleoparadoxia* and *Desmostylus* (see below). Vertebral inner structure is different from that of *Behemotops* in that the trabecular orientation appears much more random and the compact cortical layer slightly thicker (Figure 7C–D).

#### Desmostylus hesperus (Figure 8)

**Figure 8:**
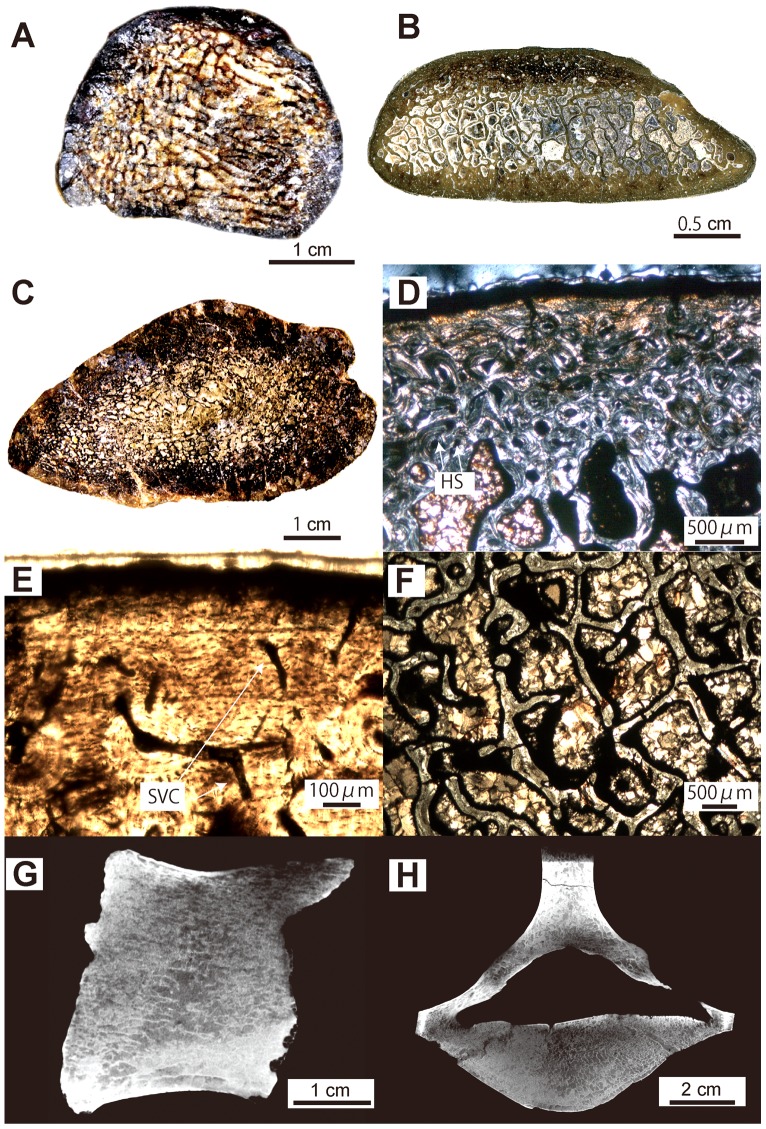
*Desmostylus hesperus* histological and microanatomical features (rib: UHR 18466 and GSJ F07745-4; femur: GSJ F07748-1; vertebra: GSJ F07743). A–C, mid-shaft section of ribs and femur. A, whole section of 6^th^ rib from an adult (UHR 18466). B, whole section of anterior thoracic rib from a juvenile (GSJ F07745-4). C, whole section of femur from an adult (GSJ F07748-1). D, cortex of B in polarized light (PL). E, detail of the primary cortical bone matrix of D in natural light (NL). SVC; simple vascular canal. F, trabeculae in the core of the section B in PL. Periphery of the bone at the top. G–H, 2^nd^ lumbar vertebra (GSJ F07743). E, virtual mid-sagittal section of the centrum. F, transverse section.

All examined bones of *Desmostylus* show a rather cancellous inner structure (Figure 8A–C). This is particularly true for the adult rib sections, whose layer of compact cortical bone is extremely thin (Figure 8A), as compared to that of the other desmostylians examined. There is no open medullary cavity (Figure 8C). Most of the section consists of a rather loose spongiosa. It must be pointed out that the humerus of the adult specimen (UHR 18466; that could not be included in the quantitative analysis because of the feeble quality of the scan) shows a much more spongy organization and a much thinner compact cortical bone than the juvenile one. Like for the humerus, the femur of the adult displays a tighter spongiosa (higher trabeculae number – smaller intertrabecular spaces) than that of the juvenile.

In both the rib and the femur of the juvenile specimens, primary bone tissue in the cortex consists of parallel-fibred bone (Figure 8D–E). The vascular network of simple vascular canals has a reticular organization. LAGs are seen in both the rib and the humerus. Remodeling is intense (as shown by the Haversian systems) and especially extensive in the rib. The trabeculae of the spongiosa are highly remodeled, but remnants of primary bone are often observable in their core (Figure 8D, F).

Only vertebrae from a juvenile specimen (GSJ F07743) were available for study (Figure 8G–H). They show, as for the other desmostylians sampled, a cancellous organization. However, the trabecular network appears much looser and the surrounding layer of compact bone is extremely thin. It cannot be determined if this feature is a result of ontogeny (as has been observed in squamate vertebrae; cf. [52]) or a characteristic of this taxon.

### Comparative Materials

**Figure 9:**
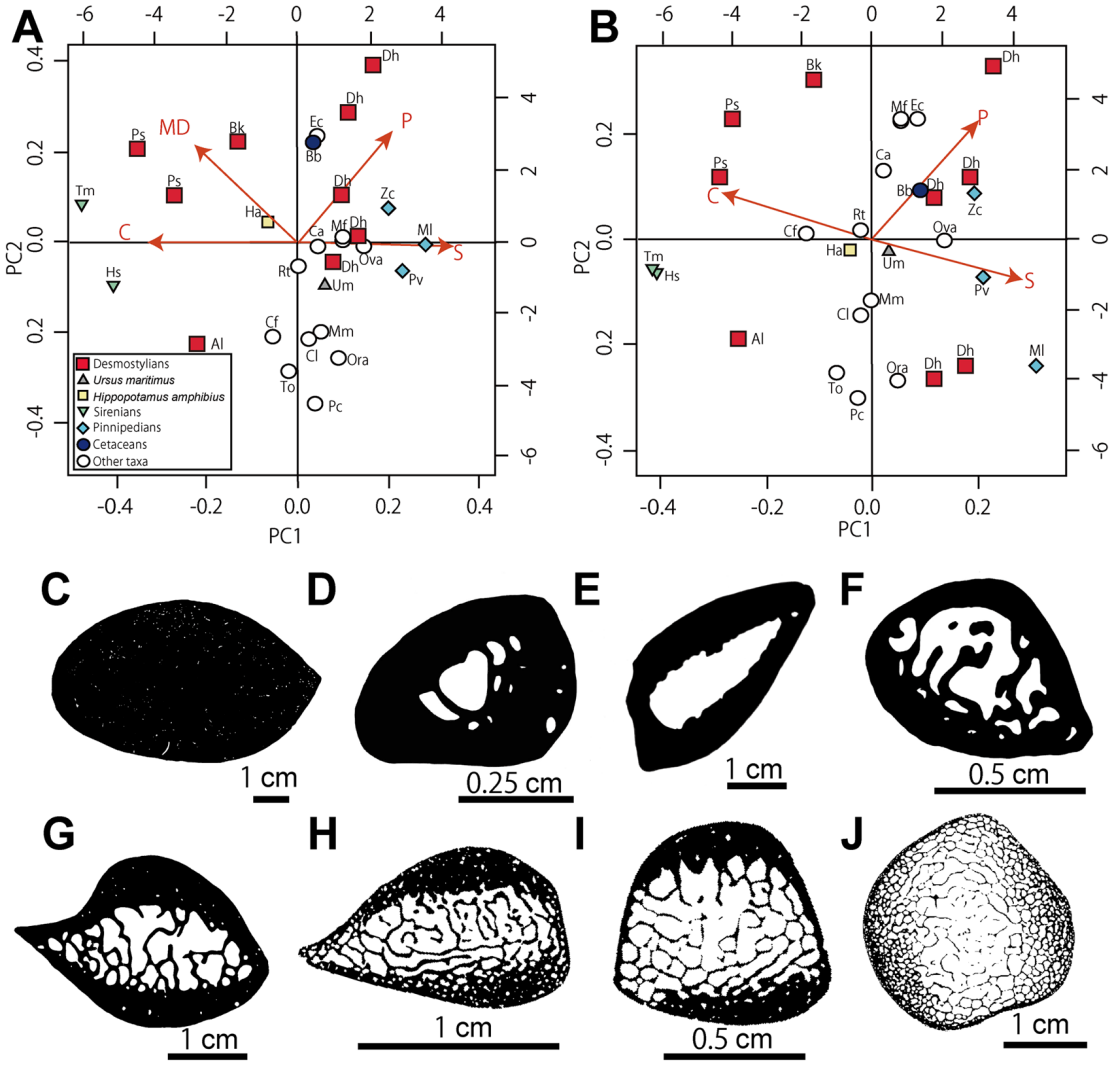
Microanatomical clusters of ribs obtained by Principal Component Analysis (PCA). A–B, graphs showing the distribution of the variance in all taxa examined according to the PCA1 and PCA2 axes. A, based on all parameters: C, bone compactness; P, proportional to the size of the medullary cavity; S, width of the transition zone between the cortical bone and the medullary region; and MD, maximum diameter of cross section. B, without the MD parameter. In red arrows are represented the vectors of the microanatomical parameters whose coordinates on the PC are the projections of their eigenvalues. Abbreviations for the taxa in the PCA graphs are described in Tables 1 and 2. C, section of *Trichechus manatus* (manatee). D, section of *Castor fiber* (beaver). E, section of *Hippopotamus amphibius* (hippopotamus). F, section of *Meles meles* (European badger). G, section of *Ursus maritimus* (polar bear). H, section of *Zalophus californianus* (California sea lion). I, section of *Phoca vitulina* (harbor seal). J, section of *Mirounga leonina* (elephant seal).

**Figure 10:**
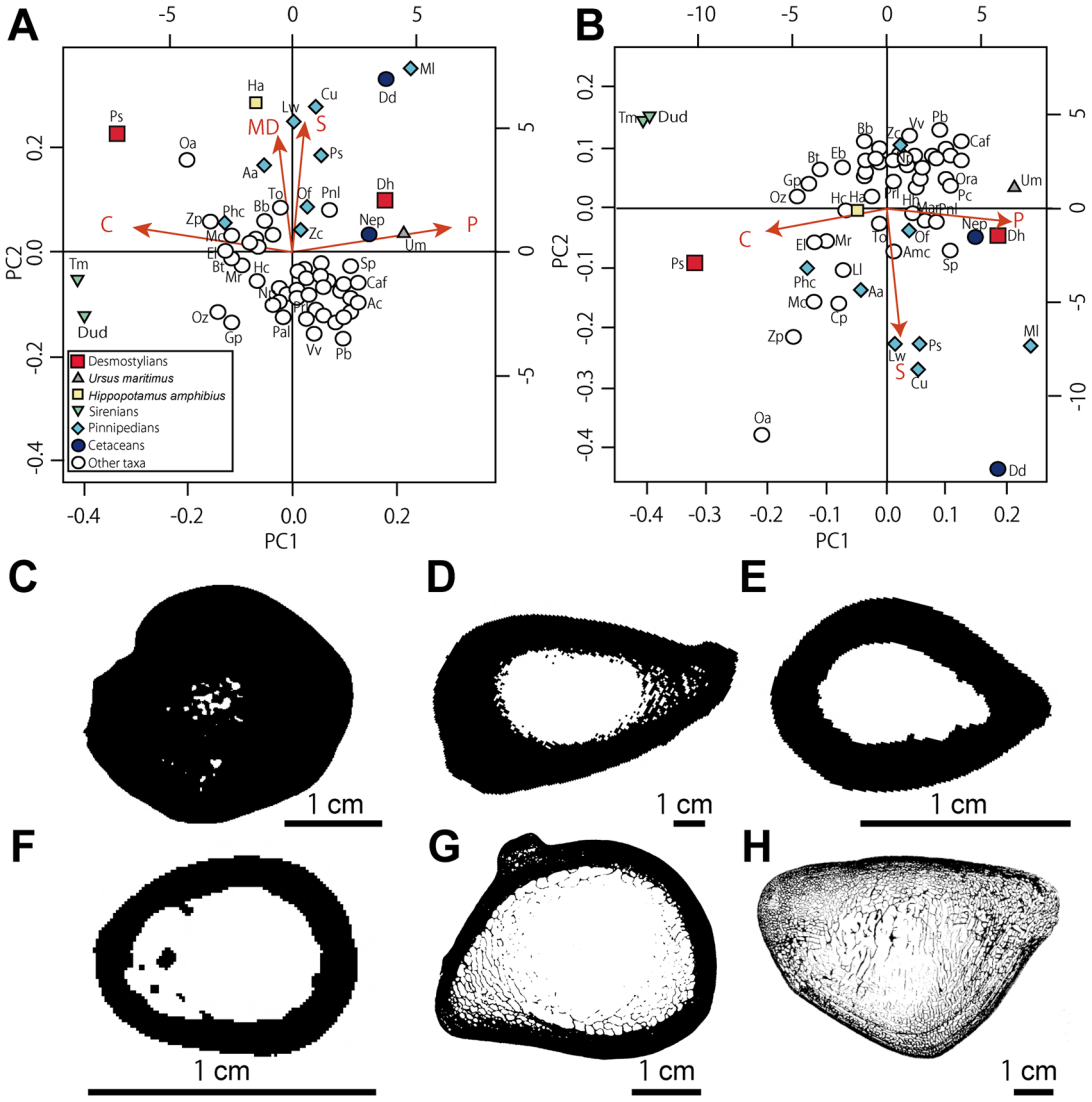
Microanatomical clusters of humeri obtained by PCA. A–B, graphs showing the distribution of the variance in all taxa examined according to the PCA1 and PCA2 axes. A, based on all parameters. B, without the MD parameter. Abbreviations of parameters are written in figure 9. Abbreviations for the taxa in the PCA graphs are described in Tables 1 and 3. C, section of *Trichechus manatus* (manatee). D, section of *Hippopotamus amphibius* (hippopotamus). E, section of *Procyon lotor* (raccoon). F, section of *Nyctereutes procyonoides* (raccoon dog). G, section of *Ursus maritimus* (polar bear). H, section of *Mirounga leonina* (elephant seal). This image (H) is from [74].

**Figure 11:**
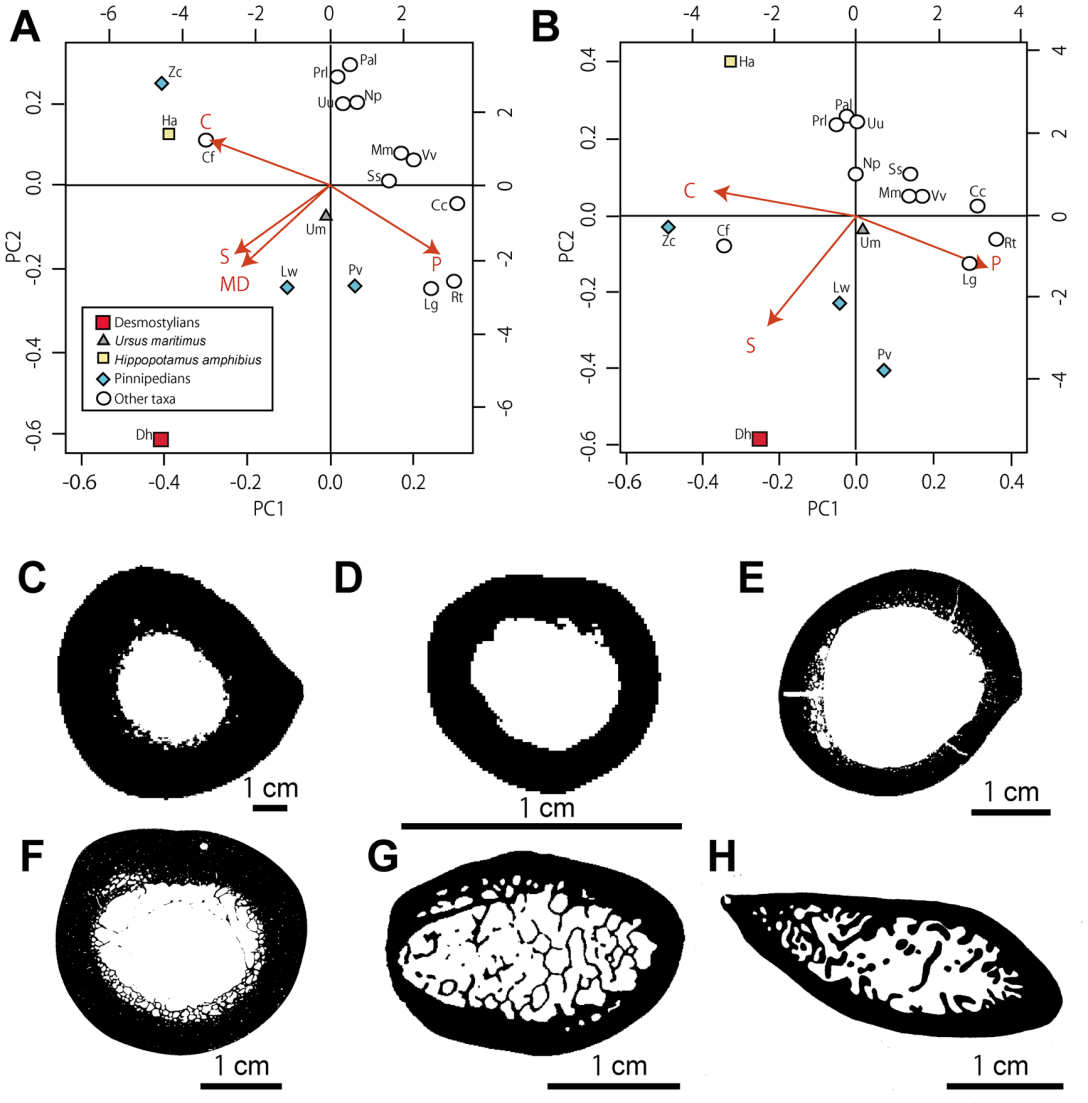
Microanatomical clusters of femora obtained by PCA. A–B, graphs showing the distribution of the variance in all taxa examined according to the PCA1 and PCA2 axes. A, based on all parameters. B, without the MD parameter. Abbreviations of parameters are written in figure 9. Abbreviations for the taxa in the PCA graph are described in Tables 1 and 4. C, section of *Hippopotamus amphibius* (hippopotamus). D, section of *Nyctereutes procyonoides* (raccoon dog). E, section of *Lama guanicoe* (guanaco). F, section of *Ursus maritimus* (polar bear). G, section of *Phoca vitulina* (harbor seal). H, section of *Leptonychotes weddelli* (Weddell seal).

**Figure 12:**
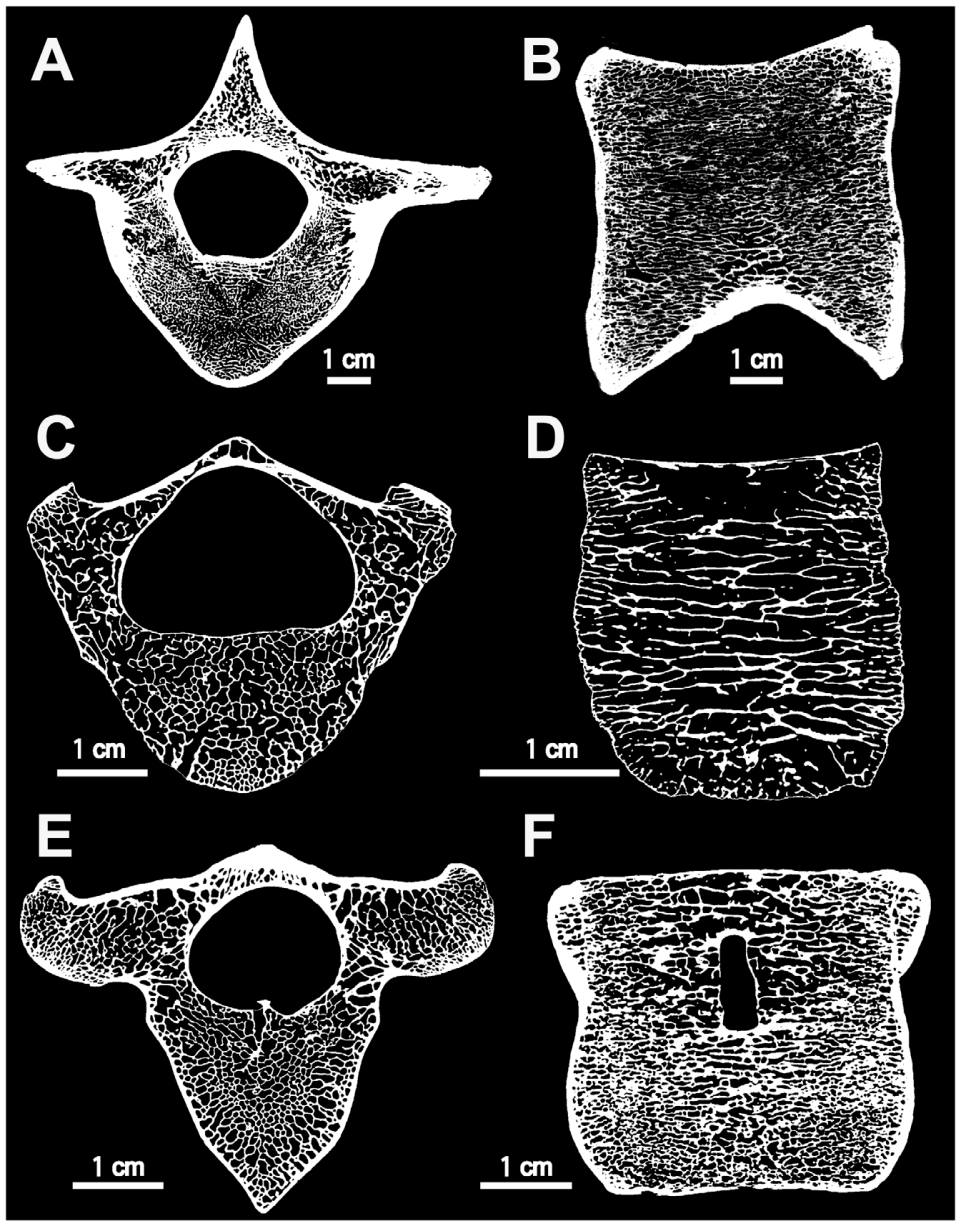
Microanatomy of thoracic vertebrate in extant mammals. A, *Trichechus manatus* (manatee). B, *Hippopotamus amphibius* (hippopotamus). C, *Phoca vitulina* (harbor seal). D, *Zalophus californianus* (California sea lion). E, *Rangifer tarandus* (caribou). F, *Ursus maritimus* (polar bear). A, C and E, virtual transverse section. B, D and F, virtual mid-sagittal section of the centrum.

Concerning both ribs and humeri, three main conditions are observed in the microanatomical structure: 1) highly compact bones with no true medullary cavity (e.g. in *Trichechus manatus* [manatee]; Figures 9C, 10C); 2) tubular bones, with a rather thick cortex and a large open medullary cavity (e.g. in *Nyctereutes procyonoides* [raccoon dog]; Figures 9E, 10D–F); 3) spongy bones, with at least most of the cortex and the whole medullary area consisting of spongiosa (e.g. in *Mirounga leonina* [elephant seal]; Figures 9G–J, 10H). The femoral samples showed only two main conditions: the tubular (Figure 11C–F) and spongy (Figure 11G–H) types. Intermediary states are of course observable between these three main conditions, with notable variations in the thickness of the compact cortical layer and of the trabeculae (e.g. much thicker cortical bone in *Hippopotamus amphibius* [hippopotamus] than in *Lama guanicoe* [guanaco]), and in the relative area of the medullary region that is spongy. Three main conditions are observed in the microstructure of the vertebrae: 1) bones with thick layers of compact bone surrounding the neural canal and the whole bone and a spongiosa with a tightly packed trabecular network (especially in *Trichechus manatus*, but also, to a lesser extent, in *Hippopotamus amphibius*; Figure 12A–B); 2) extremely thin surrounding layers of compact bone and wide spongiosa with a loose trabecular network (e.g. in all pinnipeds we examined Figure 12C–D); 3) an intermediate state relative to both the thickness of the surrounding compact layers and tightness of the trabecular network (in the terrestrial taxa we analyzed; Figure 12E–F).

### (b) Statistical Analysis

#### Cortical Development Index (CD)

Variations of the CD values are detected among desmostylian ribs (Figure 4). The CD values in most desmostylian ribs vary between 0.11 and 0.18. The values are higher in *Ashoroa* ribs, varying between 0.16 and 0.26. In each desmostylian skeleton, the CD value decreases from the 4^th^ to the 8^th^ rib.

#### Phylogenetic Significance

The phylogenetic significance of the four quantitative characters (C, MD, P and S) used for the statistical analysis was tested. The combination of the four parameters for the ribs showed no phylogenetic signal (with a probability of 0.09, H0 was accepted). However, the phylogenetic signal was tested independently for the four parameters since the value of probability was rather low. It revealed a clear absence of phylogenetic significance for P and S (probabilities of 0.87 and 0.77 respectively) and a strong phylogenetic signal in C and MD (probabilities of 0.0005 and 0.0001 respectively).

The humerus showed a phylogenetic significance for the whole tree (probability of 0.0082), with C being the only parameter with no phylogenetic signal (probability of 0.39 versus 0.005 for P, 0.0018 for S and 0.0001 for MD).

Concerning the femur, the whole tree does not show a phylogenetic signal (probability of 0.1269). A phylogenetic signal is only found for S and MD (probabilities of 0.0001 and 0.0181 respectively versus 0.29 and 0.67 for C and P respectively).

### Results of the PCA

#### Rib

The analysis on ribs (Figure 9A) shows the two main axes of the PCA explaining 82.3% of the variance (61.6 and 21.2% respectively). The first axis mainly discriminates based on S and C (projections of 0.34 and −0.33 respectively). It clearly separates sirenians and all desmostylians except *Desmostylus* from the other taxa. This is due to the strong compactness of their ribs and the absence of true medullary cavity, which confers them a compactness profile that is subhorizontal rather than S-shaped (with Bone Profiler), so that the P and S values have no real significance. Pinnipeds are also clearly separate from the others, because of their low compactness and spongy inner structure. On this axis, *Desmostylus* shows values similar to those of terrestrial taxa but closer to those of pinnipeds, like for the cetacean and the polar bear, rather than closer to those of the other desmostylians, like the hippopotamus. The second axis exclusively discriminates based on MD and P (projections of 0.23 and 0.21 respectively). *Ashoroa* separates from *Behemotops* and *Paleoparadoxia* probably because of its smaller medullary area. A second analysis without taking the size into consideration (Figure 9B; explaining 96.1% of the variance) better distinguishes *Desmostylus* from the terrestrial taxa (except *Ovis aries* [sheep] that shows peculiar microanatomical features). *Desmostylus* appears rather closer to the aquatic taxa.

#### Humerus

The two main axes of the PCA (Figure 10A) explain 73.5% of the variance (44.8 and 28.7% respectively). The first axis almost exclusively discriminates based on C and P (projection of −0.30 and 0.30 respectively). The graph clearly shows how sirenians and, to a lesser extent, *Paleoparadoxia* separate well from the other taxa. This result is linked to the presence of strong osteosclerosis and the absence of true medullary cavity in these taxa. The nearest taxon from the comparative sample is *Ornithorhynchus* (platypus), which are clearly distinguished from the others. Conversely, *Ursus maritimus* (polar bear), *Mirounga*, *Desmostylus*, and *Delphinus delphis* (common dolphin) show the strongest contrary trend, as a result of the spongy organization of their humerus. The second axis essentially distinguishes based on S (projection of 0.24) and MD (projection of 0.21). The larger sections with the widest transition zone between the cortical bone and the medullary cavity (e.g. *Mirounga*, *Delphinus*, *Callorhinus* [northern fur seal], and *Hippopotamus*) show the highest values. A second analysis, without MD was conducted and explains 94.1% of the variance (Figure 10B). Both analyses isolate *Desmostylus* from most of the comparative taxa and group it with *Neophocaena phocaenoides* (finless porpoise) and *Ursus maritimus*.

#### Femur

For the femur, the two main axes explain 88.9% of the variance (62.9 and 25.9% respectively). The first axis correlates with the four variables with a relatively high intensity (projections of −0.28, 0.25, −0.22, and −0.20 for C, P, S and MD respectively). The second axis discriminates based on MD, P, S and, to a lesser extent, C (projections of −0.19, −0.16, −0.16 and 0.11 respectively). The graph clearly separates *Desmostylus* from the other taxa, probably as a result of its spongy organization and absence of medullary cavity (Figure 11A). The combination of the two analyses (with and without MD; the second graph explaining 98.9% of the variance; Figure 11B) highlights that it is S, as a result of the spongy bone inner organization, that essentially distinguishes aquatic taxa (or of the compact inner structure for the semi-aquatic *Castor fiber* [beaver]) from the others, and *Desmostylu*s much more than pinnipeds.

### Results of the LDA

**Figure 13:**
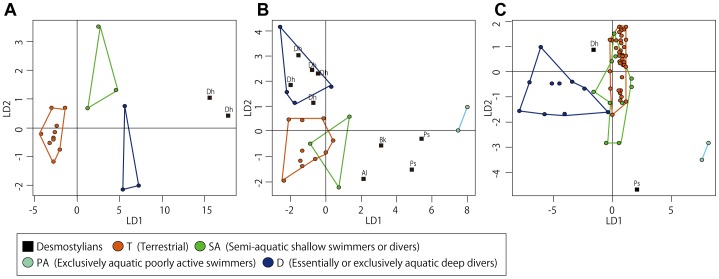
Result of the Linear Discriminant Analyses (LDA) performed on A- femora, B- ribs and C- humeri. LD1 and LD2: first and second discriminant axis, respectively. Polygons represent the limits of the various ecological categories for comparative materials (see Tables 2–4). Abbreviations for the desmostylian taxa like in Tables 1.

It must be pointed out that, considering the relative small size of our sample and resulting very small number of specimens for some lifestyle categories, the results described above have to be considered with caution. This analysis was very efficient for the femur (for which the category ‘exclusively aquatic poorly active swimmers ‘[PA] was not represented). It correctly attributed the habitat for 14 (over 16) taxa (88%–100% of the ‘Terrestrial’ [T], 70% of the ‘semi-aquatic shallow swimmers or divers’ [SA] and ‘essentially or exclusively aquatic deep divers’ [D]). The lifestyle of *Desmostylus* was clearly inferred as D. However, the graphical observation of the distribution of the taxa along the two first linear discriminant axes showed *Desmostylus*, although much closer to D than to any other category, clearly outside of D. For the rib and the humerus, the results were much more ambiguous, especially as a result of the difficulty to discriminate the SA, who were highly overlapping with T and D. The habitat was correctly attributed only for 60% (12 over 21–91% of the T, 0% of the SA, 50% of the D, 0% of the PA) and 81% (51 over 63–95% of the T, 30% of the SA, 70% of the D, 100% of the PA) of the taxa, for the rib and humerus respectively. For some categories represented by very few taxa, one wrong attribution could have a strong negative impact on the percentage values given above. The rib was the least discriminant bone in our study. For ribs, the PA category could not even be discriminated at all based on the two specimens available. For these two bones, the lifestyle of the desmostylians could not be inferred reliably. However, the graphical observation of the distribution of the taxa along the two first linear discriminant axes showed desmostylians outside of the T and SA groups. *Desmostylus* is intermediary between D and the T and SA groups for the humerus and within the D category for the rib. The other desmostylians are distributed between T and SA from the one hand and PA from the other hand (Figure 13).

## Discussion

### Morphological Differences in Desmostylian Ribs

The cortical development index (CD) was significantly higher in the ribs of *Ashoroa* (0.16–0.26) than in those of the other desmostylians (0.11–0.18; see Figure 4). Buffrénil et al. (2010) [30] previously reported that CD values higher than 0.18 characterize a pachyostotic condition. *Ashoroa* ribs (at least from the 4^th^ to the 6^th^ thoracic ones) can therefore be considered as pachyostotic. The 8^th^ rib of *Behemotops* also shows a relatively high CD value (0.16; cf. Figure 4). However, as no more anterior rib is preserved, and as CD values appear higher in ribs anterior to the 8^th^ in the other desmostylians (cf. Figure 4), it seems highly probable that *Behemotops* ribs are also pachyostotic, although it cannot be proven yet. Pachyostosis in aquatic mammals is only known in some basal cetaceans (e.g. [28], [53]) and sirenians [30], which are highly aquatic mammals. Therefore, the presence of pachyostosis in *Ashoroa's* ribs strongly suggests a fully aquatic mode of life for this taxon.

### Phylogenetic Signal

MD appears to have a phylogenetic significance in the three bones analyzed. This parameter is mainly an indicator of size. But it also provides information about the general shape of the bones by describing the thickness of a rib and the width of the midshaft of a long bone. The fact that the other parameters show a phylogenetic significance only for some bones and not for all is rather surprising. This might result from the fact that, although these parameters dealing with the bone microanatomical features and size show some degree of phylogenetic significance (which is in accord with previous studies; see e.g. [31], [45], [54]), the latter is probably rather limited. It seems indeed that the functional requirements are the main drivers of bone microanatomical features.

### Microanatomical Patterns of Desmostylians

Ribs and long bones of *Behemotops*, *Paleoparadoxia* and *Ashoroa* display a particularly dense inner organization with an extremely compact and thick cortex and a compact medullary region. Except maybe for the femur of *Behemotops,* there is no true medullary cavity but rather a relatively narrow zone with several irregularly-shaped cavities of various sizes separated by thick trabeculae. The medullary area of *Ashoroa's* ribs is almost completely filled. This difference between *Ashoroa* and the other two taxa (*Behemotops* and *Paleoparadoxia*) probably explains the differences observed in the PCA (Figure 9A–B and see above). All these three taxa are thus osteosclerotic. The microanatomical features in their ribs and long bones are intermediate between those of sirenians and some semi-aquatic taxa like *Ornithorhynchus anatinus*; they show a compact structure (cf. Figures 5A, 6A, D, 7A, 9C–D, 10C). Particularly, the ribs of *Ashoroa* are similar to those of *Trichechus manatus* in having a completely-filled medullary cavity and a pachyostotic condition. *Desmostylus* ribs and long bones, on the other hand, show a rather cancellous inner structure. There is no open medullary cavity but a loose spongiosa with thin trabeculae, surrounded by a rather thin compact cortical layer. The microanatomical features of *Desmostylus* are similar to those of some pinnipeds and cetaceans we examined (Figures 8A–C, 9I–J, 10H, 11G).

Concerning vertebrae, *Ashoroa* shows features (a relatively thick surrounding layer of compact bone and tightly packed spongiosa; cf. Figure 7C–D) similar to those observed in *Trichechus manatus* (Figure 12A). Conversely, *Behemotops* vertebrae (with their tight trabecular network but rather thin cortex) are more similar to those of *Ursus maritimus* and *Rangifer tarandus* (caribou) (Figures 5E–F, 12E–F). *Behemotops* seems thus to show a more ‘terrestrial-like’ condition in its vertebral microanatomy. *Desmostylus* vertebral features (extremely thin surrounding layer and relatively loose spongiosa; cf. Figure 8G–H) are similar to those of pinnipeds (Figure 12C–D). Two trends, which differ from what is observed in extant terrestrial taxa, are observed among desmostylian vertebrae. This is consistent with the results obtained from long bones and ribs, and thus in accordance with two distinct adaptations for an aquatic life among desmostylians.

### Bone Histological Patterns of Desmostylians

Primary periosteal bone shows similar histological features in all the desmostylian bones analyzed. It consists of parallel-fibred bone displaying cyclical growth marks, which is similar to previous reports made on mammalian histology (see [30], [55]–[57]). Various processes can be responsible for osteosclerosis (A.H; pers. obs.). In *Behemotops*, *Paleoparadoxia* and *Ashoroa*, there seems to be an excess of secondary bone deposits during remodeling, which fills the intertrabecular spaces, and thus confers on the bones a very high compactness. Osteosclerosis in these taxa appears thus driven by the same processes as in sirenians (cf. [30]). Given that *Ashoroa*'s ribs also display pachyostosis (see above), this taxon thus displays pachyosteosclerosis. These results illustrate a similar evolutionary trend between various desmostylian and sirenian ribs in the acquisitions of both osteosclerosis and pachyosteosclerosis through their evolutionary history (see [30]). Conversely, only *Desmostylus* (based on the material we analyzed) displays a spongy inner organization. This appears linked to an intense resorption activity in both the cortical and medullary regions.

### Comparisons with Other Mammals

Qualitative comparisons and the results of the PCA provide a basis to discuss lifestyle of these taxa based on their microanatomical features. Concerning ribs, *Behemotops*, *Paleoparadoxia* and *Ashoroa* are clearly distinct from terrestrial taxa as a result of their high compactness and reduced medullary cavity (Figure 9A–B). They are close to but do not group with sirenians, which display much higher compactness indices and more reduced medullary cavities. The closest taxa (sirenians apart) are semi-aquatic ones (particularly *Hippopotamus amphibius* and *Castor fiber*). However, they are different from the desmostylians in exhibiting a large open medullary cavity. *Desmostylus* ribs conversely exhibit values close to those of pinnipeds and cetaceans. Their bone microanatomical features characteristically show a relatively thin cortex (especially in the rib of the adult) and a thick spongiosa with loosely arranged thin trabeculae, and the absence of an open medullary cavity.

Concerning the humerus, *Paleoparadoxia* is characterized by a notably high compactness and a small medullary cavity. It is intermediary, again, between sirenians and *Ornithorhynchus anatinus* (Figure 10A–B), which is semi-aquatic [58]. *Desmostylus* is close to *Ursus maritimus* and a cetacean (*Neophocaena phocaenoides* [finless porpoise]). In the latter, bone microanatomical features are characterized by a relatively thinner cortex and a spongiosa with loosely arranged trabeculae. However, whereas the *Ursus maritimus* humerus displays a large open medullary cavity, *Neophocaena* and *Desmostylus*, like some pinnipeds, lack it.

Concerning the femur, *Desmostylus* does not group with any extant taxa, but the closest taxa are *Phoca vitulina* (harbor seal) and *Leptonychotes weddelli* (Weddell seal) (Figure 11A–B). The bone microanatomical features of these pinnipeds and of *Desmostylus* are characterized by a rather thin compact cortex, a thick spongiosa with loosely arranged thin trabeculae, and the absence of a large open medullary cavity. There is no significant microanatomical difference between these taxa.

The results of the PCA show *U. maritimus* close to aquatic taxa concerning the humerus, and intermediary between aquatic and terrestrial taxa concerning the femur. Despite its morphological features, which do not show particular adaptation for swimming, *U. maritimus* thus displays microanatomical features close to those of active swimmers in its limb bones, particularly the humerus. However, it must be pointed out that, as opposed to these aquatic taxa, *U. maritimus* long bones still display a true medullary cavity. This result, and notably the apparently stronger adaptation of the humerus for an aquatic mode of life, is probably linked to its swimming style because *U. maritimus* uses the forelimbs as the main propulsors during swimming [59].

Desmostylians seem to display similar microanatomies in their fore- and hindlimbs, which suggest a similar involvement in swimming by all the limbs. This hypothesis contradicts a previous study, which, based on morphological data, suggested that *Desmostylus* was a forelimb-dominated swimmer [14]. Results of the LDA are consistent with those of the PCA and reflect the same ecological trends.

### Aquatic Adaptations of Desmostylia

Previous researchers have proposed distinct hypotheses concerning the mode of life of desmostylians, although they mainly diverged in the interpretation of the degree of adaptation to an aquatic life of these taxa as a whole. Our histological study manages, for the first time, to highlight the variability in ecological patterns within desmostylians, which probably also played a role in the difficulty of understanding the ecology of these taxa. Some researchers proposed that desmostylians were more skillful in swimming than in terrestrial locomotion, like living pinnipeds (e.g. [11], [18]). Conversely, some of them suggested that they were bottom walkers like hippopotamids (e.g. [12]–[13], [20]). Domning (2002) [17] and Gingerich (2005) [14], on the other hand, regarded desmostylians as slow, heavy, quadrupedal terrestrial or semi-aquatic animals like polar bears. Barnes and Domning (2006) previously evoked different ecologies between paleoparadoxiids and desmostylids (although with different conclusions) but in an unpublished abstract without further details [60]. Our results allow discussion of these various hypotheses.

Microanatomical features of the ribs and long bones of *Behemotops*, *Paleoparadoxia* and *Ashoroa* differ significantly from those of terrestrial taxa, as well as those of *H. amphibius* and *U. maritimus*, in showing relatively high compactness. This pattern is similar to that of sirenians, although the high compactness observed in these desmostylians remains relatively lower than that observed in sirenians. The same trend is observed in *Ashoroa* vertebrae, which are similar to those of *T. manatus*. Conversely, whereas its ribs are similar to those of *Paleoparadoxia* and *Ashoroa* (i.e. osteosclerotic), *Behemotops* vertebrae resemble those of *U. maritimus*. The thin-sections of *Paleoparadoxia* humerus and virtual sections of *Behemotops* and *Ashoroa* femora, strongly suggest a high compactness. However, whereas the medullary cavity is absent in *Ashoroa* and *Paleoparadoxia*, a reduced one seems to occur in *Behemotops*. *Desmostylus* microanatomical features are also distinct from those of extant terrestrial taxa. Ribs resemble those of some pinnipeds and cetaceans in displaying a spongy inner organization without an open medullary cavity. Long bones, although distinct from those of the extant taxa analyzed, show trends similar to those of some pinnipeds (femur) and intermediate features between those of some cetaceans, pinnipeds and *U. maritimus* (humerus). Vertebrae are similar to those of pinnipeds in being particularly lightly built. In *Desmostylus*, bone microanatomical features thus illustrate trends toward an adaptation for active swimming, via their more spongy organization and lack of a medullary cavity.

The bone microanatomical specializations of desmostylians (i.e. bone mass increase and a spongy inner organization) indicate that all desmostylians were probably predominantly, if not exclusively, aquatic. Our study shows the presence of bone mass increase (BMI; e.g. [29], [61]; here osteosclerosis and pachyosteosclerosis) in the long bones of *Behemotops*, *Paleoparadoxia* and *Ashoroa* and an increase in compactness in their vertebrae. Osteosclerosis is essentially observed in relatively slow swimmers living in shallow marine environments, either hovering slowly at a preferred depth, or walking on the bottom (e.g. [29], [61]). The presence of BMI is rather incompatible with a terrestrial mode of life and rather suggests an essentially or exclusively aquatic life. It can thus be considered that *Behemotops*, *Paleoparadoxia* and *Ashoroa* were at least essentially aquatic. It cannot be stated if they were still able to come on land for some occasions, such as giving birth. On the other hand, our study shows that *Desmostylus* displays microanatomical features (notably the spongy inner organization without an open medullary cavity) characteristic of relatively active swimmers requiring efficient swimming abilities (e.g. manoeuvrability, speed) and relying on a hydrodynamic buoyancy and body trim control [27], [29], such as cetaceans and pinnipeds. These taxa display in some of their bones an osteoporotic-like status, i.e. a non-pathological condition with a thinning of the compact cortical bone thickness, and an expansion of the marrow cavity and/or of the spongiosa [27]. The spongiosa appears however looser and the layer of compact cortex thicker in *Desmostylus* than in extant cetaceans. *Desmostylus* bone microanatomical features appear thus more similar to those of pinnipeds. These results strongly suggest that *Desmostylus* was a more active swimmer than the other desmostylians we analyzed.

These results are consistent with those from some previous morphological studies, suggesting differences in the limb morphology between *Desmostylus* and other desmostylians [21], [62]. They notably suggested that all desmostylians show some degree of aquatic adaptation in their morphology, and that *Desmostylus* is much more aquatic than the other desmostylians ([16], [63] contra [60]).

A previous isotopic study suggested that *Desmostylus* spent much of its time in water, foraging on aquatic vegetation in estuarine or freshwater environments [64]. Our microanatomical data on desmostylians suggest that *Behemotops*, *Paleoparadoxia* and *Ashoroa* lived in near-shore shallow water environments, whereas *Desmostylus* might have also lived in more open marine environments. The microanatomical pattern displayed by *Desmostylus* is only known in marine taxa; conversely, bone mass increase has also been observed in freshwater taxa: choristoderans (e.g. [29]). Although desmostylian remains are found in shallow marine sediments (e.g. [65]–[66]), a more open marine life remains possible for *Desmostylus*.

Our study concludes that all desmostylians were adapted to an aquatic life, that they were probably living in a coastal marine environment, and that only *Desmostylus* acquired abilities for a more active swimming and thus displayed a distinct mode of life.

Most desmostylians are considered herbivorous taxa feeding on sea grasses [1], [36]. This is consistent with the mode of life suggested based on the occurrence of bone mass increase. However, the peculiar microanatomical features of *Desmostylus* suggest a different feeding strategy. *Desmostylus* could have fed more at the surface, on floating vegetation; so that it would not have required to control its buoyancy negatively. However, *Trichechus*, although it also dives in shallow-water environments, often swims at the surface or just below the surface (e.g. [67]), which does not prevent it from displaying bone mass increase (see [30]). The hypothesis of a link with surface swimming in *Desmostylus* would thus be consistent only if this taxon was indeed almost exclusively swimming at the surface. It was also suggested that *Desmostylus* had a dentition pattern suggesting a more abrasive diet than other desmostylians [1] and that it might be a suction feeder feeding on invertebrates like the walrus [68]–[69]. We could wonder why increased swimming abilities could have been selected in *Desmostylus* if it did not need to pursue its prey. To conclude, although it cannot yet be elucidated, the microanatomical data agree with the differences observed in *Desmostylus* dentition pattern, to suggest a different feeding strategy in this taxon, as compared to the other desmostylians.

### Bone Microanatomical Evolution among Mammalia

Through the secondary aquatic adaptation of mammalian lineages, sirenians and basal cetaceans acquired bone mass increase (e.g. [28], [30]). Conversely, recent cetaceans acquired an osteoporotic-like pattern (e.g. [27], [29]). An evolutionary shift in bone microanatomy from bone mass increase to an osteoporotic-like pattern, although observed in some extinct groups of marine reptiles (e.g. mosasauroids [70]–[71] and plesiosaurs [72]), has so far only been documented in cetaceans among mammals (e.g. [26], [28]). Our study shows a trend toward this change in osseous specialization in *Desmostylus* and so reveals that such a shift from a highly compact to a spongy inner organization also occurred in the evolutionary history of desmostylians.

## Conclusions

1. Desmostylian bone microanatomical features clearly show that they were essentially aquatic.
2. Two types of adaptation to an aquatic life are observed within desmostylians. Indeed, while bone microanatomical features in *Behemotops*, *Paleoparadoxia* and *Ashoroa* are relatively similar to those of sirenians, and suggest an adaptation to shallow marine environments, either hovering slowly at a preferred depth or walking on the bottom, those of *Desmostylus* resemble those of pinnipeds and suggest adaptation for a more active swimming.
3. Desmostylians are, with cetaceans, the second mammal group showing a shift in bone microanatomical specialization in their evolutionary history, from bone mass increase (in *Behemotops*, *Paleoparadoxia* and *Ashoroa*) to a spongy inner organization (in *Desmostylus*).

## Supporting Information

Text S1 **Institutional abbreviations appearing in the inventor numbers of specimens.**
(DOC)

Text S2 **Consensual phylogenetic tree illustrating the relationships between the taxa sampled for the study of the rib.** Modified from [4], [47]–[51].
(TIF)

Text S3 **Consensual phylogenetic tree illustrating the relationships between the taxa sampled for the study of the humerus.** Modified from [4], [47]–[51].
(TIF)

Text S4 **Consensual phylogenetic tree illustrating the relationships between the taxa sampled for the study of the femur.** Modified from [4], [47]–[51].
(TIF)

## References

[1] Inuzuka N, Domning DP, Ray CE (1994) Summary of taxa and morphological adaptations of the Desmostylia. The Island Arc 3: 522–537.

[2] Inuzuka N (2000a) Primitive Late Oligocene desmostylians from Japan and Phylogeny of the Desmostylia. Bull Ashoro Mus Pal 1: 91–124.

[3] Jacobs LL, Fiorillo AR, Gangloff R, Pasch A (2007) Desmostylian remains from Unalaska Island, Aleutian Chain, Alaska. Bull Carnegie Mus Nat Hist 39: 189–202.

[4] Beatty BL (2009) New material of *Cornwallius sookensis* (Mammalia: Desmostylia) from the Yaquina Formation of Oregon. J Vert Pal 29: 894–909.

[5] Barnes LG, Goedert JL (2001) Stratigraphy and paleoecology of Oligocene and Miocene desmostylian occurrences in western Washington State, U.S.A. Bull Ashoro Mus Pal 2: 7–22.

[6] Thomas HW, Domning DP, Roeder M, Barnes LG (2006) Abst Paleoparadoxiid Workshop “Present Status of Studies on the Paleoparadoxiid Desmostylians (Mammalia: Tethytheria)”:12..

[7] Marsh OC (1888) Notice of a new fossil sirenian from California. Am J Sci 35: 94–96.

[8] Vanderhoof VL (1937) A study of the Miocene sirenian *Desmostylus* . Univ Calif Publ Bull Dept Geol Sci 24: 169–262.

[9] Ijiri S (1938) Über den Zahnkeim M/2 ( = m1/2) und das Os sacculi dentis von *Desmostylus japonicus* . Proc Imp Acad Jpn 14: 225–230.

[10] Reinhart R (1953) Diagnosis of the new mammalian order, desmostylia. The J Geol 61: 187.

[11] Repenning CA, Packard EL. (1990) Locomotion of a desmostylian and evidence of ancient shark predation. In: *Evolutionary Paleobiology of Behavior and Coevolution*Coevolution, A. J Boucot, ed. pp., 199–203, Elsevier, Amsterdam.

[12] Inuzuka N (1984) Skeletal restoration of the desmostylians: herpetiform Mammals. Mem Fac Sci Kyoto Univ Ser Biol 9 2: 157–253.

[13] Inuzuka N (2005) The Stanford skeleton of *Paleoparadoxia* (Mammalia: Desmostylia). Bull Ashoro Mus Pal 3: 3–110.

[14] Gingerich PD (2005) Aquatic adaptation and swimming mode inferred from skeletal proportions in the Miocene desmostylian *Desmostylus* . J Mamm Evol 12: 183–194.

[15] Inuzuka N (2000b) Research trends and scope of the order desmostylia. Bull Ashoro Mus Pal 1: 9–24.

[16] Inuzuka N (2000c) Aquatic adaptations in desmostylians. Hist Biol 14: 97–113.

[17] Domning DP (2002) The terrestrial posture of desmostylians. Smithsonian Contr Pal 93: 99–111.

[18] Repenning CA (1965) Drawing of *Paleoparadoxia* skeleton. Geotimes 9: 1–3.

[19] Shikama T (1966) Postcranial skeletons of Japanese Desmostylia. Pal Soc Jpn Sp Pap 12: 1–202.

[20] Inuzuka N (2009) The skeleton of *Desmostylus* from Utanobori, Hokkaido, Japan, II. Postcranial skeleton. Bull Geol Sur Jpn 60: 257–379.

[21] Inuzuka N (2011) The postcranial skeleton and adaptation of *Ashoroa laticosta* (Mammalia: Desmostylia). Bull Ashoro Mus Pal 6: 3–57.

[22] Camp CL (1952) Earth song: a prologue to history. Berkeley, University of California Press, 127 p.

[23] Halstead L (1978) The Evolution of the Mammals, Eurobooks Limited, Italy, 116p.

[24] Kürtén B (1971) The age of mammals. Weidenfeld and Nicolson, London.

[25] Savage RJG, Long MR (1986) Mammal evolution: an illustrated guide. British museum, London, 259p.

[26] Madar SI (1998) Structural adaptations of early archaeocete long bones. In: Thewissen JGM, editor. The emergence of whales. New York: Plenum. pp 353–378.

[27] Ricqlès A, Buffrénil V (2001) Bone histology, heterochronies and the return of Tetrapods to life in water: where are we? In: Mazin JM, Buffrénil V, editors. Secondary adaptation of tetrapods to life in water. München: Verlag. pp 289–310.

[28] Gray NM, Kimberly K, Madar S, Tomko L, Wolfe S (2007) Sink or swim? Bone buoyancy control in early cetaceans. Anat Rec 290: 638–653. 10.1002/ar.20533 17516430

[29] Houssaye A (2009) “Pachyostosis” in aquatic amniotes: a review. Integr Zool 4: 325–340. 21392306 10.1111/j.1749-4877.2009.00146.x

[30] Buffrénil V, Canoville A, D’Anastasio R, Domning DP (2010) Evolution of Sirenian Pachyosteosclerosis, a model-case for the study of bone structure in aquatic tetrapods. J Mamm Evol 17: 101–120.

[31] Canoville A, Laurin M (2010) Evolution of humeral microanatomy and lifestyle in amniotes, and some comments on palaeobiological inferences. Biol J Linn Soc Lond 100: 384–406.

[32] Kaiser HE (1960) Untersuchungen zur vergleichenden Osteologie der fossilen und rezenten Pachyostosen. Palaeontographica A 114: 113–196.

[33] Mitchell ED Jr (1963) Brachydont desmostylian from Miocene of San Clemente Island, California. Bull South Calif Acad Sci 62: 192–201.

[34] Mitchell ED Jr (1964) Pachyostosis in desmostylids. Geol Soc Am Sp Pap 76: 214.

[35] Beatty BL (2006) Rediscovered specimens of *Cornwallius* (Mammalia, Desmostylia) from Vancouver Island, British Columbia, Canada. PalArch J Vert Pal 1: 1–6.

[36] Domning DP, Ray CE, McKenna MC (1986) Two new Oligocene desmostylians and a discussion of tethytherian systematics. Smithsonian Cont Pal 59: 1–56.

[37] Reinhart RH (1959) A review of the Sirenia and Desmostylia. Univ Calif Publ Geol Sci 36: 1–146.

[38] NagaoT (1935) *Desmostylus mirabilis* sp. nov. from Keton in Sakhalin. J Geol Soc Jpn 42: 822–824.

[39] Inuzuka N (2006) Postcranial skeletons of *Behemotops katsuiei* (Mammalia: Desmostylia). Bull Ashoro Mus Pal 4: 3–52.

[40] Uno H, Kimura M (2004) Reinterpretation of some cranial structures of *Desmostylus hesperus* (Mammalia: Desmostylia): a new specimen from the Middle Miocene Tachikaraushinai Formation, Hokkaido, Japan. Paleontol Res 8: 1–10.

[41] Kunz HT, Wemmer C, Hayssen V (1996) Sex, age, and reproductive condition of mammals. In: Willson DE, Cole FR, Nichils JD, Rudran R, Foster MS, editors. Measuring and monitoring biological diversity, standard methods for mammals. Smithsonian Press. pp. 279–290.

[42] Chinsamy A, Raath MA (1992) Preparation of fossil bone for histological examination. Pal Afr 29: 39–44.

[43] Sander PM (2000) Long bone histology of the Tendaguru sauropods: implications for growth and biology. Paleobiology 26: 466–488.

[44] Girondot M, Laurin M (2003) Bone profiler: a tool to quantify, model and statistically compare bone section compactness profiles. J Vert Pal 23: 458–461.

[45] Germain D, Laurin M (2005) Microanatomy of the radius and lifestyle in amniotes (Vertebrata Tetrapoda). Zool Scr 34: 335–350..

[46] Maddison WP, Maddison DR (2008) Mesquite: a modular system for evolutionary analysis, Version 2.5. Available at http://mesquiteproject.org

[47] Hassanin A, Douzery E JP (1999) The tribal radiation of the family Bovidae (Artiodactyla) and the evolution of the mitochondrial cytochrome *b* gene. Mol Phylogenet Evol 13: 227–243. 10603253 10.1006/mpev.1999.0619

[48] Deméré A, Berta A, Adam PJ (2006) Pinnipedimorph Evolutionary Biogeography. Bull Am Mus Nat Hist 279: 32–76.

[49] Bardeleben C, Moore RL, Wayne RK (2005) A molecular phylogeny of the Canidae based on six nuclear loci. Mol Phylogenet Evol 37: 815–831. 16213754 10.1016/j.ympev.2005.07.019

[50] Gilbert C, Ropiquet A, Hassanin A (2006) Mitochondrial and nuclear phylogenies of Cervidae (Mammalia, Ruminantia): systematics, morphology, and biogeography. Mol Phylogenet Evol 40: 101–117. 16584894 10.1016/j.ympev.2006.02.017

[51] Meredith RW, Janecka JE, Gatesy J, Ryder OA, Fisher CA, et al (2011) Impacts of the Cretaceous Terrestrial Revolution and KPg Extinction on Mammal Diversification. Science 334: 521–524. 21940861 10.1126/science.1211028

[52] Houssaye A, Mazurier A, Herrel A, Volpato V, Tafforeau P, et al (2010) Vertebral microanatomy in squamates: structure, growth and ecological correlates. J Anat 217: 715–727. 21039477 10.1111/j.1469-7580.2010.01307.x PMC3039184

[53] Buffrénil V, Ricqlès A, Ray CE, Domning DP (1990) Bone histology of the ribs of the archaeocetes (Mammalia: Cetacea). J Vert Pal 10: 455–466.

[54] Kriloff A, Germain D, Canoville A, Vincent P, Sache M, et al (2008) Evolution of bone microanatomy of the tetrapod tibia and its use in paleobiological inference. J Evol Biol 21: 1–11. 18312321 10.1111/j.1420-9101.2008.01512.x

[55] Klevezal GA (1996) Recording Structures of Mammals: Determination of Age and Reconstruction of Life History. Balkema, Rotterdam

[56] Sander PM, Andrassy P (2006) Lines of arrested growth and long bone histology in Pleistocene large mammals from Germany: what do they tell us about dinosaur physiology? Palaeontographica A 277: 143–159.

[57] Köhler M, Marin-Moratalla N, Jordana X, Aanes R (2012) Seasonal bone growth and physiology in endotherms shed light on dinosaur physiology. Nature doi:10.1038/nature11264. 10.1038/nature11264 22763443

[58] Fish FE, Baudinette RV, Frappell PB, Sarre MP (1997) Energetics of swimming by the platypus *Ornithorhynchus anatinus*: metabolic effort associated with rowing. J Exp Biol 200: 2647–2652. 9359371 10.1242/jeb.200.20.2647

[59] Stirling I (2009) Polar Bear: *Ursus maritimus*. In: Perrin FW, Würsig B, Thewissen JGM, editors. Encyclopedia of Marine Mammals (Second Edition). Academic Press. pp. 888–890.

[60] Barnes LG, Domning DP (2006) Paleoparadoxiid paleoecology: Were they more “aquatic” than desmostylids? Abst Paleoparadoxiid Workshop “Present Status of Studies on the Paleoparadoxiid Desmostylians (Mammalia: Tethytheria)” 17.

[61] Taylor M (2000) Functional significance of bone ballastin in the evolution of buoyancy control strategies by aquatic tetrapods. Hist Biol 14: 15–31.

[62] Fujiwara S (2009) Olecranon orientation as an indicator of elbow joint angle in the stance phase, and estimation of forelimb posture in extinct quadruped animals. J Morphol 108: 1107–1121. 10.1002/jmor.10748 19378290

[63] Inuzuka N (2000d) Preliminary report on the evolution of aquatic adaptation in desmostylians (Mammalia, Tethytheria). Oryctos 3: 71–77.

[64] Clementz MT, Hoppe KA, Koch PL (2003) A paleoecological paradox: The habitat and dietary preferences of the extinct tethythere *Desmostylus*, inferred from stable isotope analysis. Paleobiology 29: 506–519.

[65] Chinzei K (1984) Modes of occurrence, geologic range and geographic distribution of desmostylians. Monograph Assoc Geological Collab Jpn 28: 13–23 In Japanese with English summary.

[66] Matsui K, Sashida K, Uematsu S (2012) A consideration on the inhabiting depth of Japanese Desmostylia. Abst 161st Ann Meet Palaeontol Soc Jpn 56 In Japanese.

[67] Marshall CD, Huth GD, Edmonds VM, Halin DL, Reep RL (1998) Prehensile use of perioral bristles during feeding and associated behaviors of the Florida manatee (*Trichechus manatus latirostris*). Mar Mamm Sci 14: 274–289.

[68] Beatty B (2004) Evidence of suction feeding in the Desmostylidae (Desmostylia, Mammalia). Abst ICVM-7: 276.

[69] Uno H, Yoneda M, Taru H, Kohno N (2008) Dietary preference of desmostylians based on isotope, microwear and cranial morphology. Abst Fifth Conference on Secondary Adaptation of Tetrapods to Life in Water 2008: 77–78.

[70] Houssaye A (2008) A preliminary report on the evolution of the vertebral microanatomy within mosasauroids (Reptilia, Squamata). In: Everhart MJ editor. Proceedings of the Second Mosasaur Meeting, pp 81–89. Fort Hays State University, Hays.

[71] Houssaye A, Bardet N (2011) Rib and vertebral micro-anatomical characteristics of hydropelvic mosasauroids. Lethaia 45: 200–209.

[72] Wiffen J, Buffrénil V, Ricqlès A, Mazin JM (1995) Ontogenetic evolution of bone structure in Late Cretaceous Plesiosauria from New Zealand. Geobios 28: 625–640.

[73] Shikama T, Ozaki H (1974) Lost Japanese Animals. Koudansha 244p.

[74] Laurin M, Canoville A, Germain D (2011) Bone microanatomy and lifestyle: A descriptive approach. C R Palevol 10: 381–402.

