# Supplementary material for: Bone Inner Structure Suggests Increasing Aquatic Adaptations in Desmostylia (Mammalia, Afrotheria)
Source: PLoS One. 2013 Apr 2;8(4):e59146. doi: 10.1371/journal.pone.0059146 (PMC3615000; doi:10.1371/journal.pone.0059146)
Supplement: Text S1 — Institutional abbreviations appearing in the inventor numbers of specimens. (DOC) [file pone.0059146.s001.doc]

**Institutional Abbreviations: AMP,** Ashoro Museum of Paleontology, Ashoro, Japan; **GSJ,** Geological Survey of Japan, Tsukuba, Japan; **IPB,** Institute for Paleontology, University of Bonn; **NSM,** National Science Museum, Tokyo, Japan; **UFGK,** Ur- und Frühgeschichte Köln, Köln, Germany; **UHR,** Hokkaido University Museum, Sapporo, Japan; **UMUT,** The University Museum, The University of Tokyo, Tokyo, Japan; **ZFMK,** [Zoologisches Forschungsmuseum Alexander](http://www.zfmk.de/) Koenig, Bonn, Germany.
