# Supplementary figures and images for: Bone Inner Structure Suggests Increasing Aquatic Adaptations in Desmostylia (Mammalia, Afrotheria)
Source: PLoS One. 2013 Apr 2;8(4):e59146. doi: 10.1371/journal.pone.0059146 (PMC3615000; doi:10.1371/journal.pone.0059146)

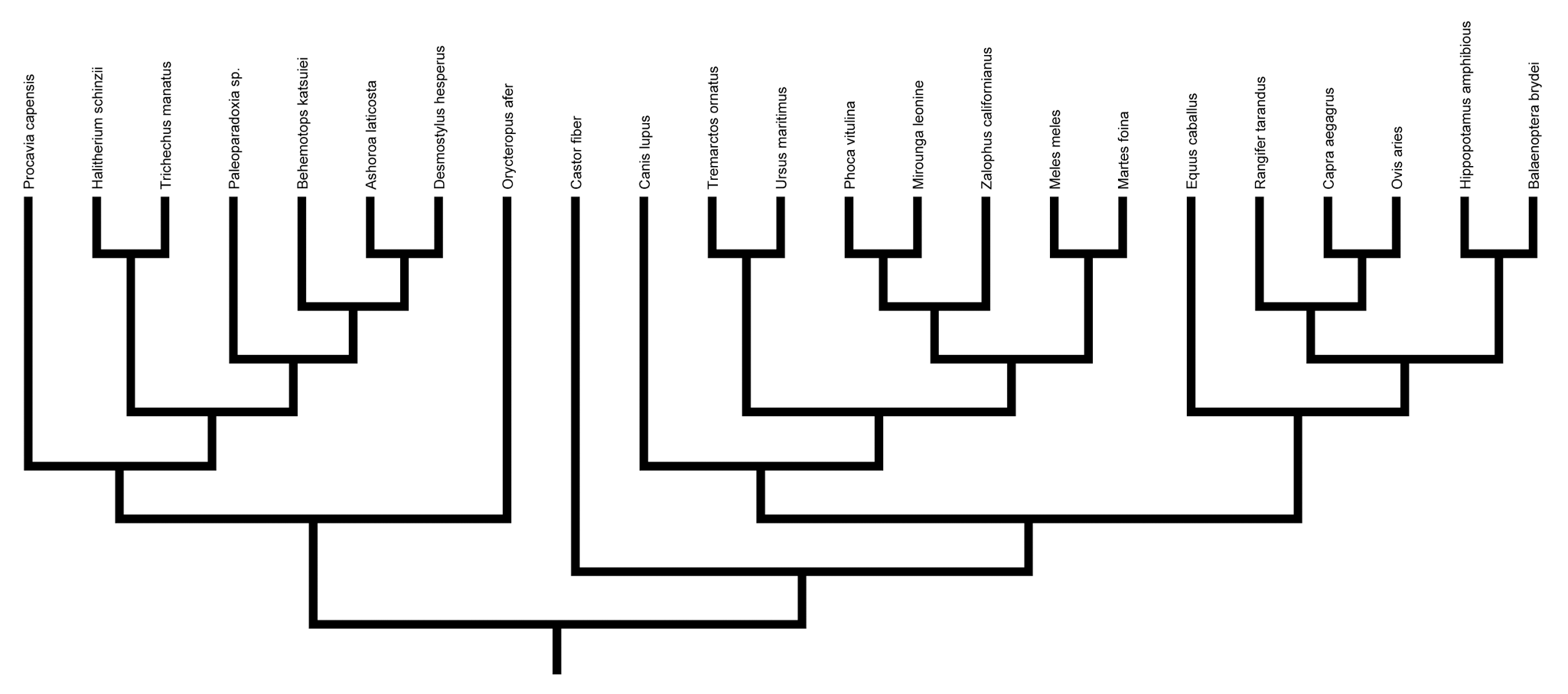

Supplement: Text S2 — Consensual phylogenetic tree illustrating the relationships between the taxa sampled for the study of the rib. Modified from [4], [47]–[51]. (TIF) [file pone.0059146.s002.tif]

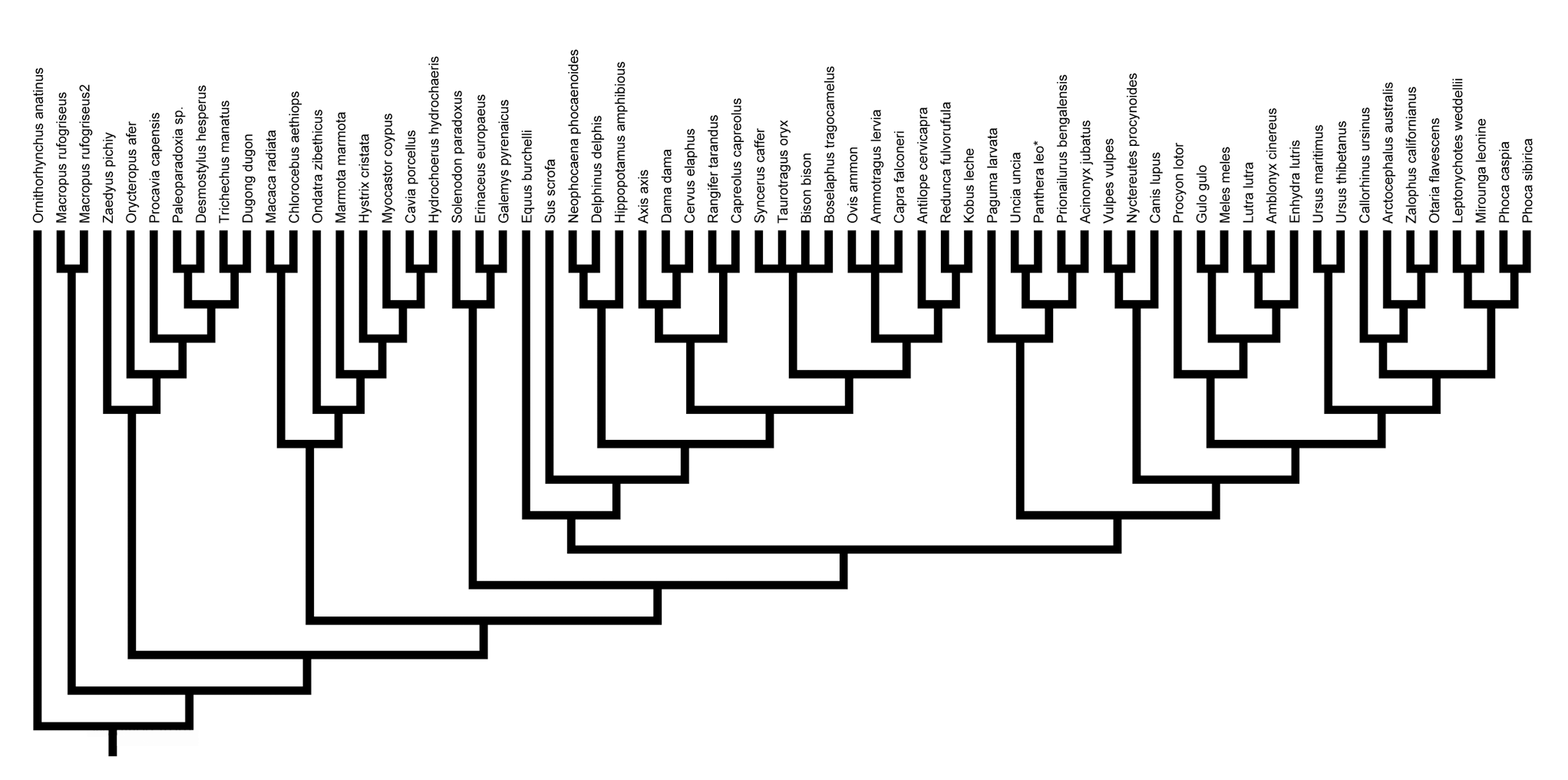

Supplement: Text S3 — Consensual phylogenetic tree illustrating the relationships between the taxa sampled for the study of the humerus. Modified from [4], [47]–[51]. (TIF) [file pone.0059146.s003.tif]

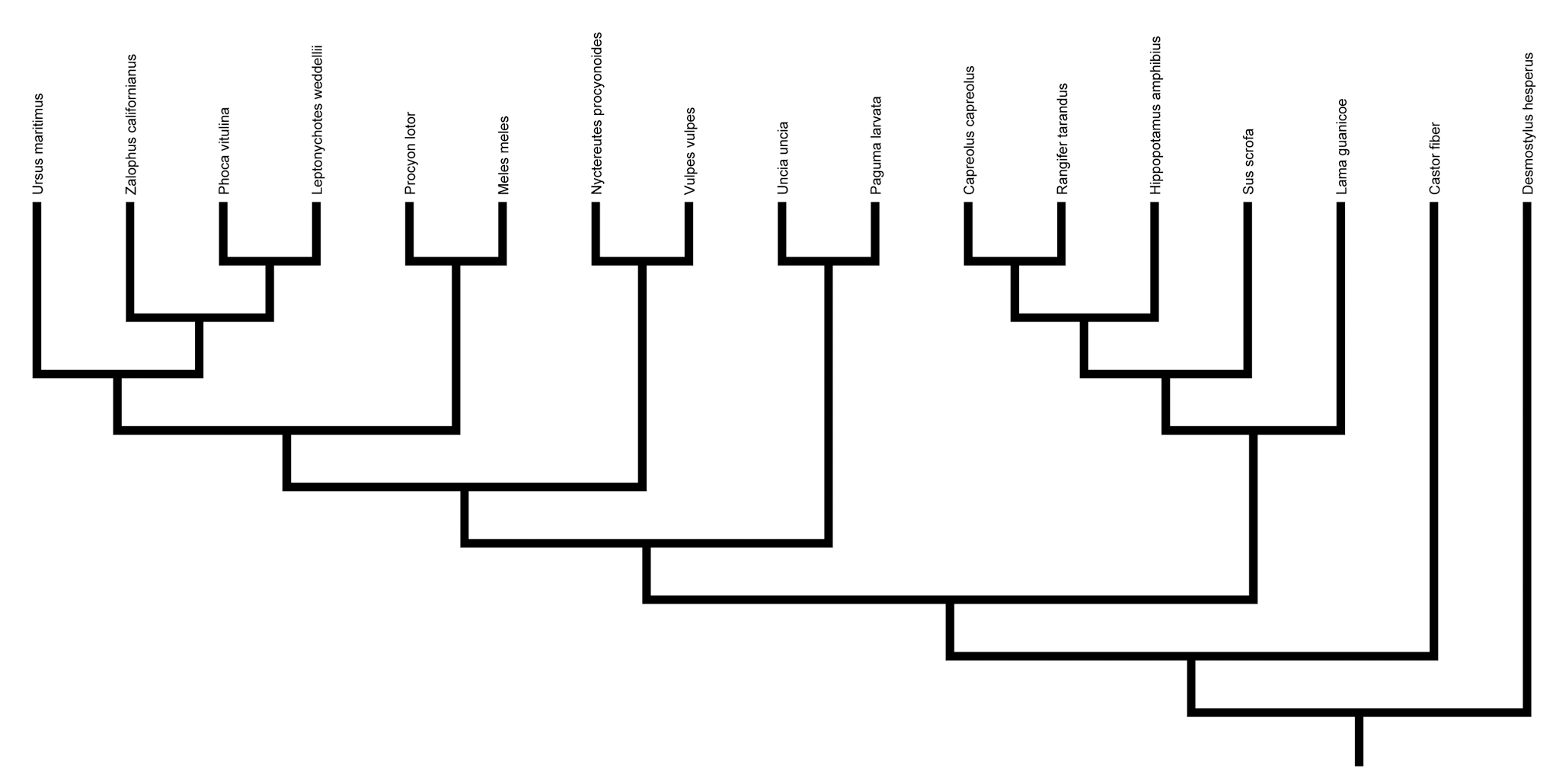

Supplement: Text S4 — Consensual phylogenetic tree illustrating the relationships between the taxa sampled for the study of the femur. Modified from [4], [47]–[51]. (TIF) [file pone.0059146.s004.tif]
